# Genetic Yield Gains and Changes in Morphophysiological-Related Traits of Winter Wheat in Southern Chilean High-Yielding Environments

**DOI:** 10.3389/fpls.2021.732988

**Published:** 2022-01-03

**Authors:** Alejandro del Pozo, Claudio Jobet, Iván Matus, Ana María Méndez-Espinoza, Miguel Garriga, Dalma Castillo, Abdelhalim Elazab

**Affiliations:** ^1^Centro de Mejoramiento Genético y Fenómica Vegetal, Facultad de Ciencias Agrarias, Universidad de Talca, Talca, Chile; ^2^CRI-Carillanca, Instituto de Investigaciones Agropecuarias, Temuco, Chile; ^3^CRI-Quilamapu, Instituto de Investigaciones Agropecuarias, Chillán, Chile; ^4^CRI-Remehue, Instituto de Investigaciones Agropecuarias, Osorno, Chile; ^5^Facultad de Agronomía, Universidad de Concepción, Chillán, Chile

**Keywords:** intercepted PAR, leaf gas exchange, NDVI, RGB images, shoot biomass

## Abstract

Both the temperate-humid zone and the southern part of the Mediterranean climate region of Chile are characterized by high wheat productivity. Study objectives were to analyze the yield potential, yield progress, and genetic progress of the winter bread wheat (*Triticum aestivum* L.) cultivars and changes in agronomic and morphophysiological traits during the past 60 years. Thus, two field experiments: (a) yield potential and (b) yield genetic progress trials were conducted in high-yielding environments of central-southern Chile during the 2018/2019 and 2019/2020 seasons. In addition, yield progress was analyzed using yield historical data of a high-yielding environment from 1957 to 2017. Potential yield trials showed that, at the most favorable sites, grain yield reached ∼20.46 Mg ha^–1^. The prolonged growing and grain filling period, mild temperatures in December-January, ample water availability, and favorable soil conditions explain this high-potential yield. Yield progress analysis indicated that average grain yield increased from 2.70 Mg ha^–1^ in 1959 to 12.90 Mg ha^–1^ in 2017, with a 128.8 kg ha^–1^ per-year increase due to favorable soil and climatic conditions. For genetic progress trials, genetic gain in grain yield from 1965 to 2019 was 70.20 kg ha^–1^ (0.49%) per year, representing around 55% of the yield progress. Results revealed that the genetic gains in grain yield were related to increases in biomass partitioning toward reproductive organs, without significant increases in Shoot DW production. In addition, reducing trends in the NDVI, the fraction of intercepted PAR, the intercepted PAR (form emergence to heading), and the RGB-derived vegetation indices with the year of cultivar release were detected. These decreases could be due to the erectophile leaf habit, which enhanced photosynthetic activity, and thus grain yield increased. Also, senescence of bottom canopy leaves (starting from booting) could be involved by decreasing the ability of spectral and RGB-derived vegetation indices to capture the characteristics of green biomass after the booting stage. Contrary, a positive correlation was detected for intercepted PAR from heading to maturity, which could be due to a stay-green mechanism, supported by the trend of positive correlations of Chlorophyll content with the year of cultivar release.

## Introduction

Since the green revolution, the yields of wheat and other cereals have increased considerably in many regions of the world, including Chile (Calderini and Slafer, 1998; Engler and del Pozo, 2013; del Pozo et al., 2014, 2019), as a result of genetic improvement and better agronomic practices. Despite these increases in world average yields over the past 50 years, the relative rates of yield increase have declined from 3.4% in 1960 to 1.3% in 2010 (Fischer et al., 2014). Nowadays, the challenge is to increase yield gains and crop yields to feed a world population of above 9 billion by 2050 (Ray et al., 2012; Fischer et al., 2014). In wheat, various reports have highlighted the importance of increasing yield potential in high-yielding areas (Parry et al., 2011; Reynolds et al., 2011; Ray et al., 2012; Hawkesford et al., 2013), which will hopefully reach 20 Mg ha^–1^.

Chile is the second-highest bread consumer globally, with 98 kg of bread consumed per capita. Thus, wheat production is of great importance, being the dominant crop in terms of the planted area (average 220.000 ha in the last 3 years), 92% of them representing bread wheat (*Triticum aestivum*) with a production of 1.5 million tons (Oficina de Estudios y Políticas Agrarias [ODEPA], 2017, 2020). It has the widest distribution of any crop in the country, covering diverse climatic regions from the semiarid Mediterranean-type climate (∼350 mm of rain) of the northern zone to the temperate-humid climate (∼2,000 mm rainfall) of the southern zone. Winter wheat is principally grown in the south part of Chile, where annual rainfall is >1,200 mm (Matus et al., 2012). The southern central area (from Ñuble to Araucanía regions) represents around 86% of national planting, with an average area over the last 3 years of 176,000 ha (Oficina de Estudios y Políticas Agrarias [ODEPA], 2017, 2020). The average national yield is 6.1 t ha^–1^, but the potential yield in many areas is much higher, particularly in temperate-humid zones where wheat can attain very high yields (>15 t ha^–1^), probably due to genotype yield potentials and the remarkable soil and climatic conditions. Thus, this unique genotype-by-environment interaction in southern Chile constitutes a unique combination for studying yield progress and the relative contribution of genetic improvement to increases in wheat yield potential.

The wheat breeding programs of INIA (Instituto de Investigaciones Agropecuarias) began in 1964, and, since then, cultivars of bread wheat (*Triticum aestivum*) and durum wheat (*Triticum durum*) adapted to the different agroclimatic zones of the country have been released. For example, the winter wheat breeding program started early in southern Chile in INIA Carillanca in 1959, first using genetic material from the global wheat collection and some introductions from France. The segregating materials were derived from F1 hybrids created in 1959, which included advanced lines containing the dwarf genes of Norin 10. Moreover, the highest grain yields genetic gains recorded globally (246 kg ha^–1^ year^–1^ or 2.6%) were for winter wheat in Chile in the humid Mediterranean climate zone (36° S; Biobío Region) due to both genetic and agronomic improvements (Matus et al., 2012; Tshikunde et al., 2019).

Potential yield (i.e., the yield achieved when the best available technology is used) has increased almost linearly since the 1960s, particularly in more favorable environments where soil water availability is not limited (Fischer and Edmeades, 2010). The yield can be determined as a function of the incoming photosynthetic active radiation (PARi) intercepted by the crop, from emergence (e) to harvest (h), and shoot dry weight (W) accumulation and distribution:


$$\text{GY}=\sum_{e}^{h}\left(fix\mathrm{PARi}\right)x\mathrm{RUE}\mathrm{xHI}$$

where GY is grain yield, *fi* is the fraction of PAR intercepted by the crop, RUE (g MJ^–1^) is the radiation use efficiency, and HI is the harvest index (= GY/W) (Reynolds et al., 2011; Fischer et al., 2014; Sadras et al., 2016).

Further increases in grain yield in environments with optimal agronomic conditions can be achieved through genetic improvement and the acquisition of various traits, such as early plant vigor, higher total crop biomass, and radiation use efficiency, increase in harvest index, and stronger stems and root systems to prevent lodging under high-yielding conditions (Foulkes et al., 2011; Parry et al., 2011; Reynolds et al., 2011).

Several studies on the genetic progress of wheat have indicated that breeding programs have achieved substantial increases in grain yield in high-yielding environments since 1960 (e.g., Shearman et al., 2005; Zhou et al., 2007; del Pozo et al., 2014; Foulkes et al., 2016; Yao et al., 2019). The genetic improvement in grain yield was closely related to increases in harvest index, spike number m^–2^, and the number of kernels per spike (e.g., Zhou et al., 2007; del Pozo et al., 2014; Gao et al., 2017), as well as increases in shoot biomass (Gao et al., 2017), leaf photosynthesis (Zheng et al., 2011), and the delayed onset of flag-leaf senescence (Foulkes et al., 2016).

Total crop biomass can be increased by improving photosynthetic capacity and/or efficiency (Reynolds et al., 2011). Surprisingly, only a small number of studies have analyzed the genotypic variability or the genetic progress in photosynthetic capacity (Zheng et al., 2011; Xiao et al., 2012; Driever et al., 2014; Carmo-Silva et al., 2017) and RUE in wheat (Acreche et al., 2009; Sadras et al., 2012; Bustos et al., 2013). A study conducted in 64 wheat genotypes grown in field conditions in England has shown natural variations in the photosynthetic capacity of the flag leaf, total biomass, and grain yield. However, no consistent correlation was found between photosynthetic capacity and grain yield (Driever et al., 2014). Recently, Carmo-Silva et al. (2017) have reported that flag leaf photosynthetic traits correlated significantly and positively with grain yield for the same wheat panel. Changes in pre-anthesis RUE of between 1.54 and 2.68 g MJ^–1^ have been observed for wheat cultivars released between 1958 and 2007 in Australia (Sadras et al., 2012). Also, a set of doubled haploid (DH) lines of spring bread wheat in southern Chile produced >15 t ha^–1^, which is 54% above the check cultivars due to their higher RUE (29% higher than the checks) during the post-anthesis period, which increased the kernels number and fruiting efficiency of the DH lines (Bustos et al., 2013).

Higher RUE post-anthesis can be attained in plants with delayed leaf senescence or the stay-green phenotype. Indeed, genotypic variability has been detected in chlorophyll content (measured with a portable leaf chlorophyll meter SPAD; Spectrum Technologies Inc., Plainfield, IL, United States) during the grain-filling period and in the rate of leaf senescence (Lopes and Reynolds, 2012). Overall, the wheat genotypes with functional stay-green characteristics showed higher grain yield (Blake et al., 2007; Chen et al., 2010).

The well-known normalized difference vegetation index (NDVI) and other indices derived from multispectral radiometers that incorporate the reflectance from the near-infrared (NIR) have been used to estimate the leaf area index and crop biomass (Gitelson et al., 2002; Royo and Villegas, 2011), and to phenotype canopy senescence or stay-green in cereals (Lopes and Reynolds, 2012; Rebetzke et al., 2016; Sadras et al., 2019). The NDVI also gives a complete canopy view of the stay-green effect and can be more informative than the SPAD index. However, the precision of vegetation indices using NIR reflectance is known to be affected by: (1) the saturation pattern at moderate to high leaf area index or biomass values, which decrease NDVI sensitivity to detecting variations between genotypes (Gitelson et al., 2002; Nguy-Robertson et al., 2012; Elazab et al., 2016); and (2) other artefactual factors that decrease the NIR reflectance, such as plant canopy structure, the contribution of soil reflectance to total canopy reflectance, and reduction of actively reflecting leaf layers at certain crop stages (Gitelson et al., 2002; Elazab et al., 2015, 2016).

Recently, the RGB-derived vegetation image indices have been proposed to replace spectral reflectance-derived indices, especially those using NIR reflectance (Hunt et al., 2013; Casadesús and Villegas, 2014; Elazab et al., 2015, 2016; Kefauver et al., 2017). Although these indices have a small spectral range (i.e., a visible spectrum governed by pigment content and composition), they have an excellent spatial resolution for quantification of green biomass (Casadesús et al., 2007; Elazab et al., 2015, 2016; Kefauver et al., 2017). Thus, they could overcome the previously mentioned limitations of NIR reflectance-based indices (Elazab et al., 2015, 2016). Moreover, reflectance relationships with pigment content remain quantitatively similar in leaves of different plant species (Gitelson et al., 2002).

There is not too much information available about the genetic progress of winter wheat in the humid temperate zone of southern Chile, where the highest yields have been reported (Matus et al., 2012; Jobet et al., 2015, 2017). In addition, it is not well studied how carbon assimilation, radiation interception, and total plant biomass changed with genetic improvement in winter cultivars in Chile. Thus, we carried out different field experiments named (a) the yield potential and (b) the genetic progress trials in high-yielding environments of central-southern Chile during the 2018/2019 and 2019/2020 seasons. In addition, we analyzed the yield progress using historical data of a high-yielding environment from 1957 to 2017 in Carillanca (southern Chile). The objectives of this work are: (1) to analyze the yield potential and progress and genetic progress of winter bread wheat in high-yielding environments in southern Chile and (2) identify changes in agronomic and morphophysiological traits of cultivars released by the National Institute of Agriculture Research (INIA) over the last 60 years.

## Materials and Methods

### Experimental Sites and Plant Material

Two field experiments were conducted in high-yielding environments of central-southern Chile, named (a) the potential yield and (b) genetic progress trials. In addition, a set of historical data from the winter breeding program of the INIA was used to analyze yield progress (Table 1). In the potential yield trials, four elite cultivars and four advanced lines from the INIA winter wheat breeding program were evaluated at seven high-yielding sites located in the southern part of the Mediterranean region of Chile (Santa Rosa, Yungay, and Humán) and the temperate-humid zone (Curacautín, Galvarino, Carillanca, and Mafil) in 2018 and 2019 (Table 1). In the southern part of the Mediterranean zone of Chile, the mean annual temperature range was 12.6–13.3°C, and precipitation was 632–1,139 mm (Supplementary Table 1); precipitation was lower in 2019, particularly at Humán, but the three sites received irrigation in both years. In the temperate-humid zone, the mean annual temperature range was 9.8–11.9°C. Galvarino was the site with the lowest precipitation (679 and 834 mm in 2018 and 2019, respectively) but received irrigation, while Carillanca (925 and 1,400 mm), Curacautin (1,856 and 1,488 mm), and Mafil (1,729 and 1,302 mm) had higher amounts of rainfall (Supplementary Table 1). For the sites that received irrigation, furrow (in Santa Rosa, Yungay, and Humán) and sprinkler (in Galvarino) irrigation of about 50 mm per application was applied three to four times after heading (when required). In all sites, soils were derived from volcanic ashes (Andisol) of low bulk density (∼0.9 g cm^–3^), large rooting depth (>100 cm), high water storage capacity and hydraulic conductivity, and high organic (>12%) contents, but also high phosphorus retention (Matus et al., 2014; Fleige et al., 2016). Plots consisted of four rows of 2 m in length and 0.20 m between rows. The seed rate was the equivalent of 200 kg ha^–1^. Seeds were disinfected using 250 cc per 100-kg seeds of Real^®^ Top (BASF; 166.6-g L^–1^ thiophanate-methyl, 8.3-g L^–1^ pyraclostrobin, and 83.3-g L^–1^ triticonazol) and 120 cc per 100-kg seeds of Punto 600 FS (ANASAC Chile; 600-g L^–1^ imidacloprid). Fertilizer additions to the plots included 250 kg ha^–1^ of triple superphosphate (46% P_2_O_5_) before sowing and 230 kg ha^–1^ of urea (46% N) applied at four leaves (Z1.4 of Zadoks stage; Zadoks et al., 1974), tillering (Z2.4), and first node (Z3.1). In addition, 100 kg ha^–1^ of sulpomag (22% K_2_O, 18% MgO, and 22% S) and 100 kg ha^–1^ of potassium muriate (60% KCL) were applied to tillering. Weeds were controlled with the application of 1 L ha^–1^ of Bacara Forte, Bayer (120-g L^–1^ flufenacet, 120-g L^–1^ flurtamone, and 120-g L^–1^ diflufenican) preemergence, and a further application of 4 g ha^–1^ of Ally, Dupont (600-g kg^–1^ metsulfuron-methyl) and 4 kg ha^–1^ of MCPA (750-g L^–1^ MCPA-dimethylammonium) post-emergence. The experimental design was a complete block with four replicates.

**TABLE 1:** A summary of experiments, sites, and evaluations.

Condition	Yield potential	Yield progress	Genetic progress	
Genotypes	Maxwell, Rocky, Kiron, Chevignon, Linea 26, Linea 010	Top 25 advanced lines	Pre-green revolution	Post-green revolution
	Linea 298, Linea 265	and cultivarsobtained from field experiments from 1957 to 2017	Druchamp (1965)	Melifen (1974), Manquefen (1977), Talafen (1982), Laurel (1987), Lautaro (1990), Tukan (1993), Kumpa (2002), Bicentenario (2010), Maxwell (2012), Pionero (2013), Rocky (2015), Kiron (2017), Chevignon (2019)
Sites	Santa Rosa (36°31′ S; 71°54′ W), Yungay (37°14′ S,	Carillanca (38°50′ S,	Santa Rosa (36°31′ S; 71°54′ W), Carillanca	
	72°01′ W), Humán (37.43° S; 72°24′ W),	72°25′ W)	Carillanca (38°50′ S, 72°25′ W), Mafil (39°90 S; 73°16 W)	
	Curacautin (38.40°31′ S; 71°90’ W),Galvarino		and Purranque (40°90; 73°16)	
	(38.45°31′ S; 72°74′ W), Carillanca (38°69′ S,			
	72°41′ W) and Mafil (39°70 S; 73°01′ W)			
Years	2018 and 2019	1959–2017	2018 and 2019	
Plot size	1.6 m^2^	2 m^2^	3.6 m^2^	
Experimental design	Complete block with four replicates	Complete block with four replicates	Complete block with four replicates	
Sowing date	1–17 May	1 May	1–17 May	
Evaluations	Grain yield and its components	Grain yield	-Grain yield and its agronomical components	
			-NDVI and intercepted PAR	
			-RBG derived vegetation indices	
Harvest date	20 Jan (Santa Rosa, Yungay, and Humán)/ 20 Feb (Curacautin, Galvarino, Carillanca, and Mafil)		20 Jan (Santa Rosa)/ 20 Feb (Carillanca, Mafil, and Purranque	
Average days to heading	177 (Santa Rosa, Yungay, and Humán)/ 200 Curacautin, Galvarino, Carillanca, and Mafil)		177 (Santa Rosa)/ 192 (Carillanca, Mafil, and Purranque	
Average grain filling	40–45 (Santa Rosa, Yungay, and Humán) / 60–70 (Curacautin, Galvarino, Carillanca, and Mafil)		40–45 (Santa Rosa) / 60–70 (Carillanca, Mafil, and Purranque	

*The year after the cultivar name refers to the cultivar release year.*

In the genetic progress trials, 13–14 winter wheat cultivars of pre- and post-green revolution, released in the country from 1965 and 2019, were evaluated at four high-yielding sites: Santa Rosa (in 2018/2019 and 2019/2020) in the Mediterranean climate zone under irrigated conditions, and Carillanca (in 2018/2019 and 2019/2020), Purranque (in 2018/2019), and Mafil (in 2019/2020) in the temperate-humid zone (Table 1). Each plot consisted of 9 rows of 2 m in length and 0.2 m apart, and the seed rate was 180 kg ha^–1^ (∼330 plants m^–2^). The crop management was similar to the potential yield trials. The plots were sprayed against fungal diseases with 6.25 cc L^–1^ of Juwell Top (Basf; 150-g L^–1^ fenpropimorph, 125-g L^–1^ kresoxim-methyl, and 125-g L^–1^ epoxiconazole) and 0.8 L ha^–1^ of Priori (Syngenta; 250-g L^–1^ azoxystrobin). At Santa Rosa, furrow irrigation was applied around four main developmental stages of the crop (if necessary): tillering (Z2.4), flag leaf just visible (Z3.7), early heading (Z5.0), and medium milk (Z7.5). The experimental design was a complete block with four replicates.

Data from 25 advanced lines and cultivars obtained from field experiments conducted at Carillanca from 1957 to 2017 were used for yield progress analysis. The plots consisted of five rows of 2 m in length and 0.20 m between rows. The seed rate was the equivalent of 180 kg ha^–1^. The sowing date was in May-June of each year. Crop fertilization and weed control were performed as recommended for each year, but no fungicides or insecticides were used in the trials. The experimental design was a complete block with four replicates. To estimate grain yield, an area of 2 m^2^ was harvested at maturity.

### Agronomic Traits

Data on the following were collected: (a) days to heading when 50% of the spikes, which were at stage Z5.9, were determined by regular observations of the plots; (b) plant height (from the soil level up to the ends of the awns); (c) shoot dry weight (Shoot DW) and harvest index (grain yield/Shoot DW) determined from a 1-m row harvested at maturity and placed in a forced-air oven at 60°C for 48 h; (d) the number of spikes per m^2^ was extrapolated from a 1-m row and the number of kernels per spike and the thousand kernels weight were determined in 10 spikes taken at random from the same 1-m row; (e) the number of kernels per m^2^ was calculated by multiplying the number of kernels per spike by the number of spikes per m^2^; and (f) grain yield by harvesting 1.6 m^2^ (i.e., the whole plot) and 2 m^2^ (i.e., five central rows) for the potential yield and genetic progress trials, respectively.

### Flag Leaf Traits

The leaf area and the specific leaf area were determined on three flag leaves per plot at early grain filling (Z7.0). The leaves were cut, and, immediately, the leaf area was measured with the WinDIAS Leaf Image Analysis System (Delta-T Devices, United Kingdom), and then placed in a forced-air oven at 60°C for 48 h and weighed for specific leaf area determination. Leaf gas exchange parameters, the net assimilation rate (An), and stomatal conductance (*gs*) were determined in three flag leaves per plot using a portable open system infra-red gas analyzer (CIRAS-3 model, PP Systems, Amesbury, MA, United States) with a 0.250-L min^–1^ flow rate, 400 ppm CO_2_, and leaf temperature at 25°C. Measurements were made at the heading (Z5.9), anthesis (Z6.9), and late milk (Z7.9) stages between 11:00 and 15:00 on sunny days at a photon flux density of at least 1,500 mmol m^–2^ s^–1^, using a PLC3 universal leaf cuvette (1.75 cm^2^ of the leaf area). In addition, chlorophyll content was measured using a DUALEX sensor (Dualex Scientific, Force A, France) as a non-destructive measurement.

### Vegetation Indices

The normalized difference vegetation index (NDVI) was determined with a handheld spectroradiometer (GreenSeeker, Trimble, United States) in 13–14 genotypes in the genetic progress trial in 2018 and 2019. Measurements were performed on 4–5 occasions from tillering to the grain filling stage for each environment (Z2.7, main stem and seven tillers; Z3.7, flag leaf just visible; Z4.7, flag leaf sheath opening; Z6.9, anthesis complete; and Z7.7, late milk). Measurements were performed at 60 cm above the top of the canopy. The NDVI corresponds to the differences between the reflectance (R) in the near-infrared (760 nm) and red (660 nm) bands and is calculated as (Raun et al., 2001):


$$NDVI=\left(R_{760}-R_{660}\right)/\left(R_{760}+R_{660}\right)$$

In 2019, RGB images of each plot were acquired using a DJI Mavic Pro (DJI, China) unmanned aerial vehicle (UAV) equipped with an RGB 24-mm camera with a 1/2.3″ 12 MP sensor. The flights were carried out 25 m above the ground level using Pix4D software^1^ for automated flight control. Three flights were performed at each site at booting (Z4.7), heading (Z5.9), and grain filling (Z7.7, late milk). Orthomosaics were created from RGB images using Agisoft PhotoScan Professional software (Agisoft LLC, Russia), and then individual images from each plot were obtained. The CerealScanner plugin (Shawn Kefauver, University of Barcelona^2^) of the open-source ImageJ analysis platform FIJI (Fiji is Just ImageJ^3^) was used to extract the different color parameter information from the images (Hue, Saturation, Intensity, Lightness, a *, b *, u *, v *). Also, other parameters related to the active photosynthetic canopy and senescence, such as green area (GA = pixels with 60° < Hue < 180°); and greener area (GGA = pixels with 80° < Hue < 180°) were extracted (detailed information on these parameters is found in Casadesús et al., 2007; Hunt et al., 2013; Vergara-Díaz et al., 2016; Kefauver et al., 2017).

### Intercepted PAR and Leaf Area Index

The intercepted PAR was evaluated at the same stages of the NDVI by using a 1-m long probe with 64 PAR sensors and a BF5 reference PAR sensor (SunScan canopy analyzer); three determinations at the bottom of each plot were taken by placing the 1-m sensor in parallel with the crop row between 11:00 and 16:00 h. Beer’s law was used to estimate the leaf area index:


$$I=Io\times e^{\left(-K\times LAI\right)}$$

and then equation was transformed to:


LAI=-ln(I/Io)×1/K

where LAI is the leaf area index, *Io* is the incident PAR at the top of the canopy, *I* is the transmitted PAR at the bottom of the canopy, K is the canopy extinction coefficient (calculated using the Campbell equation; more information is available in the SunScan manual^4^), and *I/Io* is the light transmittance (from heading, it included transmittance through both active and senescenced leaves). The fraction of PAR intercepted (*fi*) was measured as (Tao et al., 2018):


$$fi=1-\frac{I}{Io}$$

Later, the *fi* by each cultivar was plotted against accumulated thermal time (Tb = 0°C) from sowing to maturity at Santa Rosa and Carillanca, using measurements performed in 2018 and 2019.

The daily incoming radiation (MJ m^–2^) during 2018 and 2019 was obtained from weather stations located at Santa Rosa and Carillanca; the incident PAR (PARi) was considered to represent half of the total incident radiation. The daily intercepted PAR was calculated as [*fi* × PARi], and then the accumulated intercepted PAR (IPAR) was calculated from crop emergence to heading and from heading to maturity. RUE (g MJ^–1^) for each cultivar was estimated as Shoot DW/IPAR.

### Statistical Analysis

Differences among genotypes (G) and environments (E) were determined through ANOVA using Statgraphics Centurion Version 18.1.12 (Statgraphics Centurion, 2018). In the ANOVAs, genotypes and sites were considered fixed factors and replicate random factors. For the potential yield trials, regression analyses were performed between the grain yield BLUE value (best linear unbiased estimator) of each cultivar and the grain yield BLUE value of cultivars at each site and year (environmental index; Finlay and Wilkinson, 1963). The sites’ phenotypic correlation (Pearson) was determined using the BLUE values for each trait of the genetic progress trials. Also, regression analyses of the year of cultivar release and grain yield with the traits were performed using BLUE values for each site in the 2 years of evolution. The BLUE values were calculated by a site and across all sites using META-R (Multi Environment Trail Analysis with R) for Windows (META-R, 2016) created by the Centro Internaciónal de Mejoramiento deMaíz y Trigo (CIMMYT). It is a suite of R scripts (R Core Team, 2018) linked by a graphical user interface (GUI) designed in Java language. Figures were created by MS Excel (Microsoft Corporation, 2019).

## Results

### Productivity of Winter Wheat in Southern Chile (Potential Yield Trials)

**FIGURE 1:**
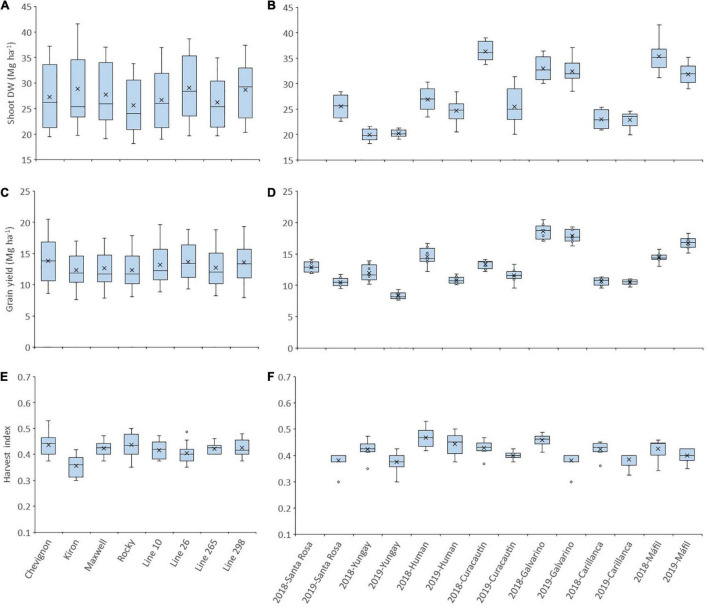
Shoot dry weight **(A,B)**, grain yield **(C,D)**, and harvest index **(E,F)** of eight winter wheat genotypes grown at seven high-yielding sites in 2018 and 2019. A box and whisker plot was created using the best linear unbiased estimator (BLUE) values for each genotype in each site. The plots show population minimum, 25th percentile/median/75th percentile and maximum, and the mean (x) for each genotype across sites and years **(A,C,E)** and genotypes for each site in the 2 years **(B,D,F)**. Circles indicate outlier data.

The shoot dry weight (Shoot DW) across the seven sites between latitudes 36°31′ S and 39°90 S and 2 years (2018 and 2019) was between 25.64 (cv. Rocky) and 29.04 Mg ha^–1^ (Line 26) (Figure 1A), but the average of all genotypes reached 36.35 Mg ha^–1^ at one site (Curacautin) in 2018 (Figure 1B). The grain yield across the seven sites and 2 years were between 12.34 (Kiron) and 13.85 Mg ha^–1^ (Chevignon), and the average of all genotypes was 17.90 and 18.58 Mg ha^–1^ at Galvarino in 2018 and 2019, respectively (Figures 1C,D). The harvest index exhibited significant (*p* < 0.001) differences among genotypes and sites in both years, but not for the genotype x site interaction (data not shown). The harvest index across the sites and years was between 0.36 (cv. Kiron) and 0.44 (cv. Chevignon), and the average of all genotypes was the highest (0.47) at Human in 2018 (Figures 1E,F).

**FIGURE 2:**
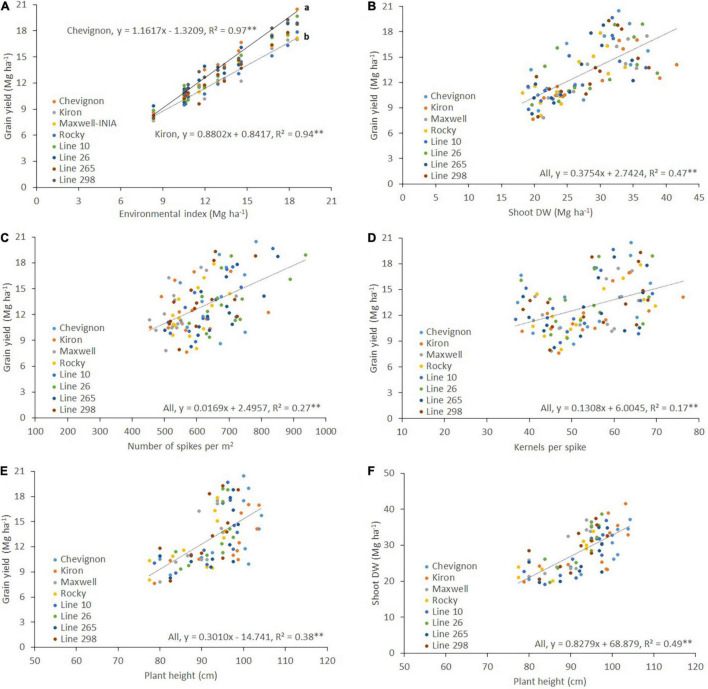
Relationships between grain yield and **(A)** environmental index, **(B)** shoot dry weight (Shoot DW), **(C)** the number of spikes per m^2^, **(D)** the number of kernels per spike, and **(E)** Plant height; and **(F)** relationship between Shoot DW and plant height of eight winter wheat genotypes grown at seven high-yielding sites in 2018 and 2019. The figures were created using the best linear unbiased estimator (BLUE) values for each genotype in each site and year. The slopes of Chevignon and Kiron in **(A)** were statistically different at *p* < 0.01 using the *t*-test.

The linear regression between grain yield and the environmental index indicated that the highest regression coefficient (Finlay–Wilkinson slope) was for cv. Chevignon (*b* = 1.16) and the lowest was for cv. Kiron (*b* = 0.88) (Figure 2A). Grain yield had a positive and significant correlation with shoot DW (*r*^2^ = 0.47, *p* < 0.01; Figure 2B). Weak but positive significant correlations were detected for grain yield with number of spikes per m^2^ (*r*^2^ = 0.27, *p* < 0.01; Figure 2C), and number of kernels per spike (*r*^2^ = 0.17, *p* < 0.01; Figure 2D). Moreover, medium significant positive correlations were detected between plant height with both grain yield (*r*^2^ = 0.38, *p* < 0.01; Figure 2E) and Shoot DW (*r*^2^ = 0.49, *p* < 0.01; Figure 2F).

### Yield Progress Analysis of Winter Wheat in Southern Chile for the 1959–2017 Period

**FIGURE 3:**
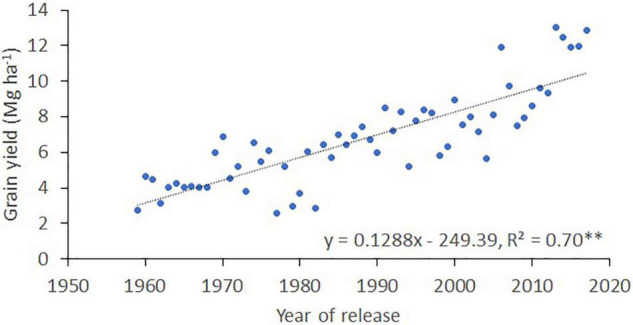
Yield progress of winter bread wheat at the INIA Carillanca (38° 41′S; 72° 25′ W) in southern Chile. The figures were created using the best linear unbiased estimator (BLUE) values for 25 cultivars and advanced lines each year.

The average grain yield of the 25 cultivars and advanced lines evaluated in southern Chile (at INIA Carillanca) between 1959 and 2017 increased from 2.7 Mg ha^–1^ in 1959 to 12.9 Mg ha^–1^ in 2017 (Figure 3); the regression analysis indicates that the rate of increase in grain yield between 1959 and 2017 was 128.8 kg ha^–1^ per year. However, analysis of the last 10 years showed much higher progress in grain yield (of 637 kg ha^–1^ per year) and stagnation of grain yield during the last 5 years at an average of 12.45 Mg ha^–1^.

### Productivity of Winter Wheat Genotypes Released Between 1965 and 2019 (Genetic Progress Trials)

#### Phenological Development and Plant Height

**FIGURE 4:**
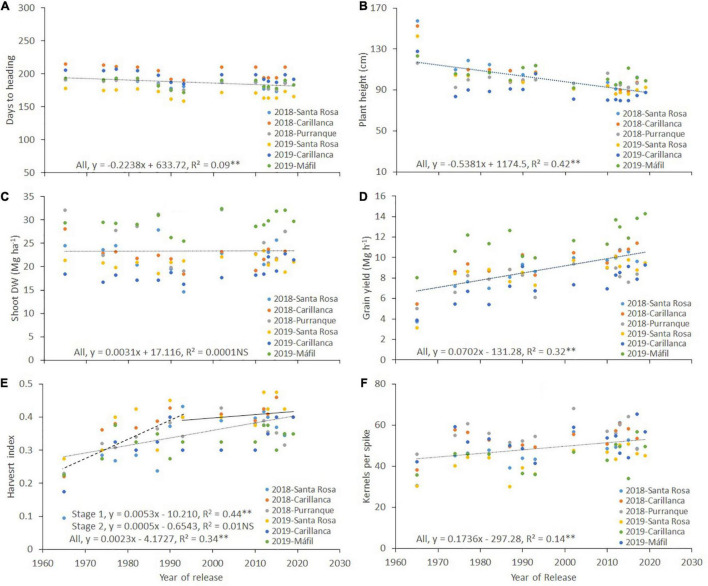
Relationships between the year of cultivar release and days to heading **(A)**, plant height **(B)**, shoot dry weight (Shoot DW) **(C)**, grain yield **(D)**, harvest index **(E)**, and the number of kernels per spike **(F)** for each wheat cultivar released in Chile between 1965 and 2019. The environments were a combination of sites and years: Santa Rosa in 2018 and 2019, Carillanca in 2018 and 2019, Purranque in 2018, and Mafil in 2019. Each data point represents the best linear unbiased estimator (BLUE) of the cultivar in each site in each year.

On average, days to the heading of the 13–14 cultivars ranged (at the three sites) from 181 to 199 in 2018 and 180 to 192 in 2019 (Table 2). Santa Rosa tended to have the least number of days to heading among the three sites (average, 184 and 169 in 2018 and 2019, respectively), and Carillanca was the highest (204 and 196 in 2018 and 2019, respectively). The correlation for days to heading among the three sites (phenotypic correlation) was >0.91 (*p* < 0.001) in both years (Table 3). Days to heading were negatively correlated (weak but significant) with the year of cultivar release (*r*^2^ = 0.09, *p* < 0.01; Figure 4A).

**TABLE 2:** Mean values of agronomic traits at three sites in 2018 and 2019, ANOVA, and Pearson correlations of the year of cultivar release and grain yield with agronomic traits.

Genotype			Days to heading^2^		Plant height (cm)		Shoot DW (Mg ha^–1^)		Grain yield (Mg ha^–1^)		Harvest index	
	2018	2019	2018	2019	2018	2019	2018	2019	2018	2019	2018	2019
Druchamp			199.1 a	192.2 a	142.1 f	131.0 f	28.2 d	23.1 a	4.7 a	5.0 a	0.18 a	0.23 a
Melifen			197.5 ab	190.3 b	102.5 cd	97.9 cd	23.0 bc	22.3 a	7.5 b	8.2 b	0.32 b	0.28 b
Manquefen			198.2 ab	192.1 a	109.6 e	99.6 cd	25.2 bcd	22.5 a	8.4 bc	9.2 bcd	0.32 b	0.37 cdef
Talafen			196.30 bc	191.6 a	109.2 e	101.0 de	23.6 bcd	22.4 a	7.9 b	8.5 b	0.33 b	0.35 cde
Laurel			191.8 d	183.9 d	105.0 cde	96.5 bcd	27.2 cd	22.2 a	8.6 bcd	9.2 bcd	0.33 b	0.33 bc
Lautaro			181.7 f	174.8 h	101.3 cd	100.0 cd	20.4 ab	22.0 a	9.3 cd	9.2 bcd	0.39 c	0.38 cdef
Tukan			182.3 ef	171.9 i	107.1 de	108.5 e	17.4 a	21.0 a	7.7 b	8.0 b	0.39 c	0.34 cde
Kumpa			195.0 c	187.1 c	94.6 ab	88.1 ab	26.1 cd	24.1 a	9.9 d	9.6 bcde	0.41 c	0.34 cd
Bicentenario			195.0 c	186.8 c	99.2 bc	91.5 abc	23.5 bcd	23.2 a	9.4 cd	9.1 bc	0.39 c	0.33 cde
Maxwell			183.7 e	178.5 f	90.8 a	87.3 ba	22.4 bc	23.6 a	9.7 cd	10.7 de	0.41 c	0.41 f
Pionero			182.5 ef	177.3 g	90.8 a	87.9 ab	22.5 bc	24.1 a	9.9 d	10.2 bde	0.39 c	0.38 def
Rocky			180.8 f	175.7 h	91.3 a	92.3 abcd	23.0 bc	24.1 a	9.7 cd	10.3 cde	0.39 c	0.39 ef
Kiron			194.5 c	187.2 c	98.8 bc	92.1 abcd	26.1 cd	24.6 a	9.8 d	10.2 cde	0.34 b	0.38 cdef
Chevignon			–	180.4 e	–	92.9 abcd	–	24.1 a	–	11.0 d	–	0.38 def
Santa Rosa			184.3 b	169.2 c	107.4 c	99.1 b	22.9 a	21.0 b	8.6 b	8.5 b	0.33 a	0.39 b
Carillanca			203.7 a	196.4 a	103.1 b	89.3 a	22.4 a	18.8 a	9.4 c	7.3 a	0.38 b	0.33 a
Purranque/Mafil^1^			183.9 b	185.1 b	99.2 a	104.4 c	26.0 b	29.5 c	7.9 a	11.8 c	0.35 a	0.33 a
**ANOVA**	**Df**											
Genotype (G)	12	13	<0.001	<0.001	<0.001	<0.001	<0.001	0.58	<0.001	<0.001	<0.001	<0.001
Site (S)	2	2	<0.001	<0.001	<0.001	<0.001	0.001	<0.001	<0.001	<0.001	<0.001	<0.001
G*S	24	26	<0.001	<0.001	<0.001	0.106	0.352	0.687	0.382	0.222	<0.001	0.016
Replicate	3	3	0.977	0.966	0.706	0.947	0.977	0.163	0.062	0.963	0.008	0.711
Year of release^2^			−0.32*	−0.28	−0.70**	−0.60**	−0.16	0.14	0.72**	0.48**	0.62**	0.55**
Grain yield^3^			0.04	−0.25	−0.73**	−0.12	−0.17	0.80**	–	–	0.82**	0.34*

*^1^Purranque in 2018 and Mafil in 2019.*

^2,3^The Pearson correlations of the year of release and grain yield were performed using BLUE values for each site in the 2 years of evaluation, *p < 0.05; **p < 0.01.

**TABLE 3:** Pairwise phenotypic correlations among sites BLUEs of the studied traits in 2018 and 2019.

	Sites comparison	Days to heading	Plant height	Shoot DW	Grain yield	Harvest index	Number of spikes per m^2^	Number of kernels per spike	Thousand kernels weight	Number of Kernels per m^2^	Leaf area	Specific leaf area	Chlorophyll content	An	*gs*
2018	Carillanca & Purranque	0.97***	0.80**	0.46	0.75**	0.82***	0.61*	0.69**	0.46	0.61*	−0.16	0.54	0.85***	0.35	0.54
	Carillanca & Santa Rosa	0.91***	0.99***	0.53	0.92***	0.82***	0.85***	0.85***	0.86***	0.84***	0.04	−0.49	0.71**	0.52	0.29
	Purranque & Santa Rosa	0.92***	0.79**	0.51	0.70**	0.77**	0.45	0.77**	0.3	0.57*	0.47	−0.22	0.56*	0.18	0.44
2019	Carillanca & Máfil	0.95***	0.77**	0.36	0.65*	0.52	0.61*	0.77**	0.88***	0.63*	0.77**	0.58*	−0.34	0.26	0.63*
	Carillanca & Santa Rosa	0.98***	0.92***	−0.26	0.71**	0.77**	−0.5	0.23	0.72**	0.01	0.79***	0.51	0.23	0.48	0.39
	Máfil & Santa Rosa	0.94***	0.75**	−0.24	0.73**	0.45	0.09	0.34	0.84***	0.33	0.55*	0.65*	0.77**	0.32	0.32

*p < 0.05; **p < 0.01; ***p < 0.001.

Plant height was significantly (*p* < 0.001) higher in cultivars released before 2000, with cv. Druchamp (>130 cm) from 1965 was the tallest (Table 2), and the effect of the site was significant (*p* < 0.001) in 2018 and 2019. The genotype x site interaction was significant (*p* < 0.001) only in 2018. The phenotypic correlation for plant height among the three sites was 0.79–0.99 in 2018 and 0.75–0.92 in 2019 (Table 3). The relationship between plant height and the year of cultivar release was negative and significant (*r*^2^ = 0.42, *p* < 0.01; Figure 4B).

#### Shoot Dry Weight, Grain Yield, and Agronomic Components

The Shoot DW and grain yield were significantly (*p* < 0.001) different among genotypes and sites in 2018 and 2019 except for the genotype effect in 2019 (Table 2). The genotype x site interaction was not significant in 2018 and 2019. The cv. Tukan (released in 1993) had the lowest Shoot DW in both years, and the highest values of Shoot DW were attained at the two most southern sites, Purranque and Mafil (Table 2). The phenotypic correlations for Shoot DW among the three sites were low and not significant (*p* > 0.05) in either year (Table 3).

Grain yield and harvest index were significantly (*p* < 0.001) different among genotypes and sites in both years, and the genotype x site interaction was only significant (*p* < 0.001) for harvest index in 2018 and 2019 (Table 2). In 2018, cvs. Kumpa (2002), Maxwell (2012), Pionero (2013), Rocky (2015), and Kiron (2017), and, in 2019, cv. Chevignon (2019) had a ∼100 and 120% higher yield than cv. Druchamp (1965), respectively (Table 2). At Mafil in 2019, cv. Chevignon reached 14.3 Mg ha^–1^ (data not shown), but the highest harvest index was cv. Maxwell (Table 2). The phenotypic correlations were, in general, high and significant for grain yield (0.70–0.92 for the three sites in 2018; 0.65–0.73 in 2019) and harvest index (0.77–0.82 in 2018; 0.45–0.77 in 2019 and significant only for Carillanca and Santa Rosa) (Table 3).

The yield components: number of spikes per m^2^, number of kernels per spike, thousand kernels weight, and number of kernels per m^2^ were significantly (*p* < 0.001) different among genotypes (except for the number of spikes per m^2^ in 2019) and sites (except for the number of spikes per m^2^ in 2018), and no significant differences were detected for the genotype x site interactions except for thousand kernels weight in 2018 (*p* < 0.01) and 2019 (*p* < 0.001) (Table 4). Phenotypic correlations for yield components were positive and significant for the number of spikes per m^2^ (0.45–0.85 except Purranque and Santa Rosa), the number of kernels per spike (0.69–0.85), and the number of kernels per m^2^ (0.57–0.84) in 2018, while, in 2019, they were only significant between Carillanca and Mafil (Table 3). The thousand kernels weight phenotypic correlation was significant (*p* < 0.01 and *p* < 0.001) in 2019 (0.72–0.88), while, in 2018, it was only significant (*p* < 0.001) for Carillanca and Santa Rosa.

**TABLE 4:** Mean values of yield components at three sites in 2018 and 2019, ANOVA, and Pearson correlations of the year of cultivar release and grain yield with yield components.

Genotype			Number of spikes per m^2^		Number of kernels per spike		Thousand kernels weight		Number of Kernels per m^2^	
	2018	2019	2018	2019	2018	2019	2018	2019	2018	2019
Druchamp			470.4 b	555.8 ab	38.2 a	36.0 a	41.0 abc	40.9 bc	17683 a	19967 a
Melifen			571.7 cd	577.2 ab	52.6 cd	48.5 bcd	38.3 ab	37.8 a	30070 de	28182 b
Manquefen			511.3 bc	544.8 ab	54.6 de	47.3	43.1 cd	41.7 cd	27792 cde	25594 b
Talafen			498.3 bc	545.8 ab	52.2 cd	47.7 bcd	40.4 abc	42.8 def	25954 cde	26123 b
Laurel			599.2 d	577.4 ab	46.8 b	43.4 abc	43.7 cd	39.5 b	28212 cde	25544 b
Lautaro			498.8 bc	584.2 ab	48.8 bc	41.4 ab	45.6 de	46.6 g	24379 bc	24457 ab
Tukan			366.3 a	522.2 a	49.1 bcd	37.9 a	51.3 f	53.8 i	18003 a	19647 a
Kumpa			510.8 bc	571.9 ab	60.1 f	51.1 cd	44.7 cd	42.4 cde	30904 e	29453 b
Bicentenario			478.8 b	599.0 b	52.1 cd	47.8 bcd	46.0 de	43.4 ef	24967 bcd	28941 b
Maxwell			382.5 a	529.2 ab	54.2 cde	49.6 bcd	49.9 ef	49.4 h	20731 ab	26083 b
Pionero			487.1 b	562.2 ab	60.7 f	48.7 bcd	37.2 a	37.9 a	29666 de	27581 b
Rocky			458.3 b	588.3 ab	58.3 ef	43.0 abc	42.5 bcd	44.2 f	26384 cde	25167 b
Kiron			474.2 b	513.7 a	50.1 bcd	56.1 d	46.1 de	43.2 def	24040 bc	28992 b
Chevignon			–	584.8 ab	–	50.4 cd	–	43.9 ef	–	29635 b
Santa Rosa			502.6 a	556.4 b	47.2 a	42.5 a	41.1 a	39.6 a	23707 a	23567 a
Carillanca			483.6 a	517.1 a	53.1 b	44.7 a	46.2 c	42.9 b	25713 ab	23076 a
Purranque/Mafil^1^			469.4 a	609.9 c	56.1 c	51.9 b	44.2 b	47.6 c	26452 b	31647 b
**ANOVA**	**Df**									
Genotype (G)	12	13	<0.001	0.163	<0.001	<0.001	<0.001	<0.001	<0.001	<0.001
Site (S)	2	2	0.122	<0.001	<0.001	<0.001	<0.001	<0.001	0.041	<0.001
G*S	24	26	0.169	0.497	0.271	0.218	0.01	<0.001	0.421	0.088
Replicate	3	3	0.017	0.065	0.153	0.083	0.339	0.23	0.297	0.002
Year of release^2^			−0.33*	0.02	0.47**	0.37*	0.25	0.18	0.09	0.29
Grain yield^3^			0.05	0.53**	0.51**	0.63**	0.35*	0.40**	0.43**	0.73**

*^1^Purranque in 2018 and Mafil in 2019.*

^2,3^The Pearson correlations of the year of release and grain yield were performed using BLUE values for each site in the 2 years of evaluation, *p < 0.05; **p < 0.01.

Shoot dry weight was not correlated with the year of cultivar release (*r*^2^ = 0.0001, *p* > 0.05; Figure 4C), but grain yield had a positive and linear relationship; the slope indicated that the genetic gain in yield was 70.2 kg ha^–1^ per year (*r*^2^ = 0.32, *p* < 0.01; Figure 4D). Harvest index and the year of cultivar release relationship were positive and significant (*r*^2^ = 0.34, *p* < 0.01; Figure 4E), but no significant increase in harvest index was observed after 1993 (Stage 1, *r*^2^ = 0.44, *p* < 0.01; Stage 2, *r*^2^ = 0.01, *p* > 0.05; Figure 4E). Also, harvest index was positively correlated with grain yield in both years (*r* = 0.82, *p* < 0.01 and *r* = 0.34, *p* < 0.05 for 2018 and 2019, respectively; Table 2).

The correlation between the number of spikes per m^2^ and the year of cultivar release was negative and significant only in 2018 (*r* = –0.33, *p* < 0.05; Table 4). The correlation between the number of kernels per spike and the year of cultivar release was positive and significant; the genetic progress for the number of kernels per spike was 0.17 kernels per year (*r*^2^ = 0.14, *p* < 0.01; Figure 4F). The correlations between grain yield and the number of spikes per m^2^, the number of kernels per spike, thousand kernels weight, and the number of kernels per m^2^ across sites were positive and significant for 2018 and 2019 except for the number of spikes per m^2^ in 2018 (Table 4).

#### Flag Leaf Traits

Leaf area, specific leaf area, and chlorophyll content were significantly (*p* < 0.05, *p* < 0.01, and *p* < 0.001) different among genotypes and sites, except for leaf area and specific leaf area in 2018 where sites have no significant effect (Table 5). The genotype x site interaction was significant for the leaf area in 2018 and 2019, while it was only significant for a specific leaf area in 2018 and chlorophyll content in 2019. Cultivars Talafen (1982) and Lautaro (1990) had the highest and lowest leaf areas. Significant differences for An and *gs* were observed among genotypes and sites in 2018 and 2019, but the genotype x site interactions were insignificant (Table 5). Cultivars with the highest An and *gs* were Manquefen (1977) and Talafen (1982), and the lowest values were recorded in Melifen (1974), Tukan (1993), and Rocky (2015).

**TABLE 5:** Mean values of flag leaf physiological traits evaluated at heading-anthesis at three sites in 2018 and 2019, ANOVA, and Pearson correlations of the year of cultivar release and grain yield with flag leaf physiological traits.

Genotype			Leaf area (cm^2^)		Specific leaf area (cm^2^ g^–1^)		Chlorophyll content		An (μmol m^–2^ s^–1^)		*gs* (mmol m^–2^ s^–1^)	
	2018	2019	2018	2019	2018	2019	2018	2019	2018	2019	2018	2019
Druchamp			34.8 abcd	25.2 c	137.5 a	166.6 f	36.4 bcde	33.5 bcd	13.8 abcd	14.7 bc	237.0 abcd	183.8 bc
Melifen			36.1 bcd	27.4 c	172.5 abc	161.2 def	34.3 b	32.4 b	10.5 a	14.1 bc	184.5 a	187.2 bc
Manquefen			39.9 de	35.0 de	134.3 a	152.8 bcd	34.7 bc	34.9 bcde	15.5 d	15.1 bc	294.2 bcd	210.0 c
Talafen			45.8 e	37.4 e	147.7 ab	152.0 bcd	35.6 bcd	35.7 cdef	15.5 d	15.5 c	312.1 d	216.0 c
Laurel			35.6 bcd	27.8 c	176.6 bc	163.2 ef	31.2 a	32.0 b	13.3 abcd	13.7 bc	309.1 bcd	179.0 abc
Lautaro			28.8 a	19.3 a	181.1 bc	164.3 f	38.7 de	32.9 bc	14.1 bcd	13.6 bc	301.8 bcd	185.1 bc
Tukan			37.9 cd	32.6 d	163.4 abc	161.1 def	30.8 a	29.0 a	11.8 abc	11.3 a	246.5 abcd	146.4 a
Kumpa			33.3 abc	21.0 ab	170.6 abc	141.6 ab	39.0 d	37.7 ef	14.1 bcd	14.4 bc	247.6 abcd	172.2 ab
Bicentenario			31.9 abc	26.1 c	147.3 ab	149.8 bc	39.1 d	38.1 f	13.1 abcd	15.5 c	228.4 abc	185.1 bc
Maxwell			34.8 abcd	26.5 c	165.2 abc	147.6 abc	39.4 d	36.2 def	15.1 cd	14.0 bc	290.8 bcd	169.3 ab
Pionero			30.1 ab	26.9 c	177.4 bc	160.6 def	38.1 de	37.0 ef	12.0 abcd	13.7 bc	218.3 bc	167.7 ab
Rocky			33.2 abc	26.7 c	191.2 c	151.3 bcd	35.6 bcde	36.4 ef	10.7 ab	13.3 b	204.0 a	165.3 ab
Kiron			32.3 abc	26.7 c	137.8 a	137.4 a	37.9 cde	37.3 ef	14.7 cd	14.5 bc	255.6 abcd	180.9 abc
Chevignon			–	24.1 bc	–	142.4 abc	–	34.9 bcde	–	13.4 b	–	176.3 abc
Santa Rosa			35.8 a	24.8 a	161.4 a	157.5 b	36.8 b	33.6 a	16.4 c	12.2 a	292.1 b	180.6 b
Carillanca			36.3 a	25.9 a	163.1 a	139.9 a	35.1 a	35.5 b	9.3 a	13.0 b	241.5 a	133.8 a
Purranque/Mafil^1^			32.8 a	31.4 b	160.7 a	163.7 c	38.1 b	35.5 b	14.3 b	17.4 c	235.7 a	237.9 c
**ANOVA**	**Df**											
Genotype (G)	12	13	<0.001	<0.001	0.002	<0.001	<0.001	<0.001	0.008	<0.001	0.002	<0.001
Site (S)	2	2	0.076	<0.001	0.954	<0.001	0.032	0.002	<0.001	<0001	< 0.001	<0.001
G*S	24	26	0.001	0.005	0.027	0.084	0.603	<0.001	0.51	0.165	0.511	0.77
Replicate	3	3	0.657	0.076	0.258	0.349	<0.001	0.014	0.338	<0.001	0.088	<0.001
Year of release^2^			−0.33*	−0.25	0.2	−0.41**	0.39*	0.37*	−0.04	−0.08	−0.13	−0.17
Grain yield^3^			−0.28	0.27	0.08	0.19	0.28	0.18	−0.15	0.55**	0.11	0.64**

*An is net photosynthesis; and gs stomatal conductance.*

*^1^Purranque in 2018 and Mafil in 2019.*

^2,3^The Pearson correlations of the year of release and grain yield were performed using BLUE values for each site in the 2 years of evaluation, *p < 0.05; **p < 0.01.

The phenotypic correlations between the three sites were significant only in 2019 for the leaf area (0.55–0.79) and the specific leaf area (0.51–0.65 except for Carillanca and Santa Rosa). For chlorophyll content, phenotypic correlations between the three sites were significant in 2018 (0.56–0.85), while it was only significant in 2019 (0.77) between Máfil and Santa Rosa. An and *gs* phenotypic correlations between the three sites for the 2 years were non-significant except for *gs* (0.63) between Carillanca and Máfil in 2019 (Table 3).

There was a trend for the leaf area (*r* = –0.33, *p* < 0.05 in 2018) and the specific leaf area (*r* = –0.41, *p* < 0.01 in 2019) to reduce with the year of cultivar release (Table 5). Cultivar Rocky (2015) exhibited the highest specific leaf area in 2018 and cv. Kiron (2017), the lowest in both years (Table 5). Chlorophyll content had a positive and significant correlation with the year of release in both years (*r* = 0.39, *p* < 0.05 in 2018; and *r* = 0.37, *p* < 0.05 in 2019), while there was no correlation with An and *gs* (Table 5). Grain yield was positively correlated with An and *gs* in 2019 (*r* = 0.55, *p* < 0.01 for An; *r* = 0.64, *p* < 0.01 for *gs*; Table 5).

#### Leaf Area Index, Intercepted PAR, Normalized Difference Vegetation Index, Chlorophyll Content, and Vegetation Indices

**FIGURE 5:**
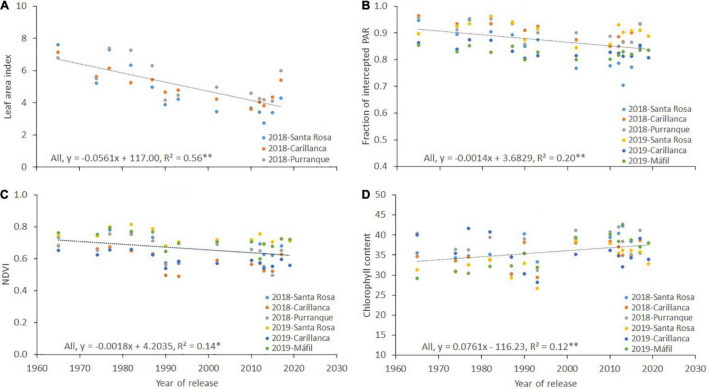
Relationship between the year of cultivar release and **(A)** leaf area index, **(B)** fraction of intercepted PAR (*fi*), **(C)** NDVI, and **(D)** chlorophyll content at Santa Rosa, Carillanca, Purranque, and Mafil in 2018 and 2019. Each data point represents the best linear unbiased estimator (BLUE) of the cultivar in each site in each year.

**FIGURE 6:**
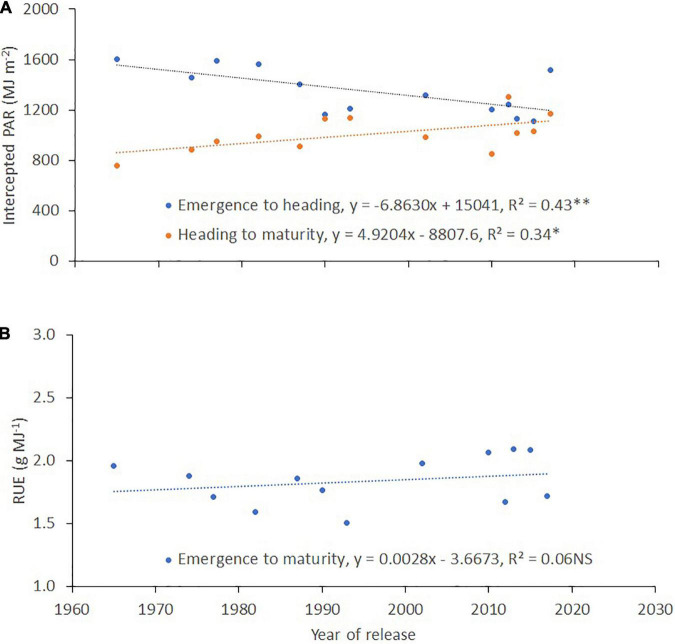
Relationship between the year of cultivar release and **(A)** intercepted PAR (IPAR) from emergence to heading and from heading to maturity, and **(B)** radiation use efficiency (RUE). Each data point represents the cultivar’s best linear unbiased estimator (BLUE) across two sites and 2 years (four environments of Santa Rosa and Carillanca in 2018 and 2019).

There was a reducing trend for the leaf area index (*r*^2^ = 0.56, *p* < 0.01; Figure 5A), the fraction of intercepted PAR (*fi*, *r*^2^ = 0.20, *p* < 0.01; Figure 5B), and the NDVI (*r*^2^ = 0.14, *p* < 0.05; Figure 5C) with the year of cultivar release at the three sites and during both years of evaluation, while the opposite was observed for chlorophyll content (*r*^2^ = 0.12, *p* < 0.01; Figure 5D). The relationship between the year of cultivar release and the intercepted PAR was negative between crop emergence and heading (*r*^2^ = 0.43, *p* < 0.01; Figure 6A) and positive from heading to maturity (*r*^2^ = 0.34, *p* < 0.05; Figure 6A). No clear trend was observed between the year of cultivar release and RUE (*r*^2^ = 0.06, *p* > 0.05; Figure 6B).

**FIGURE 7:**
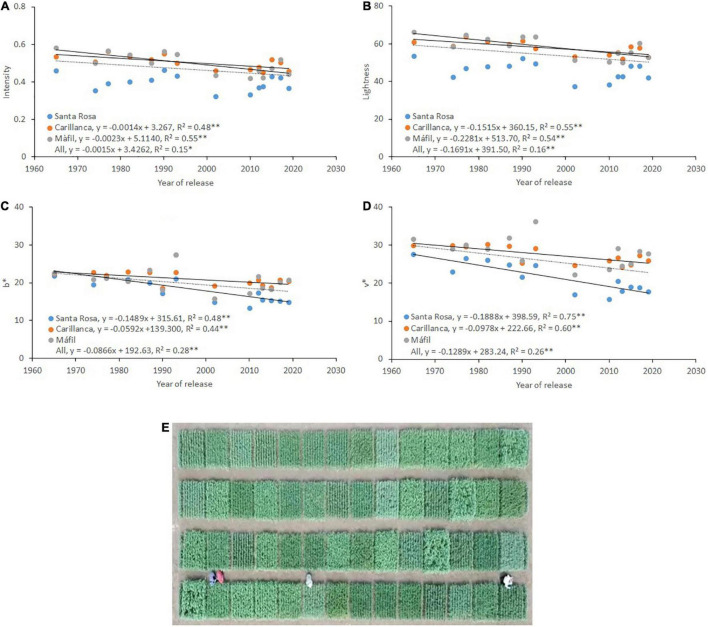
Relationship between the year of cultivar release and the RGB-derived vegetation indices: **(A)** intensity, **(B)** lightness, **(C)** b*, and **(D)** v* at Santa Rosa, Carillanca, and Mafil in 2019. An aerial view of the experimental field at Santa Rosa **(E)**. Each data point represents the cultivar’s best linear unbiased estimator (BLUE) in each site.

Also, there was a reducing trend for the RGB-derived vegetation indices: intensity (*r*^2^ = 0.15, *p* < 0.05; Figure 7A), lightness (*r*^2^ = 0.16, *p* < 0.01; Figure 7B), b* (*r*^2^ = 0.28, *p* < 0.01; Figure 7C), and v* (*r*^2^ = 0.26, *p* < 0.01; Figure 7D) measured between booting and grain filling with the year of cultivar release at the three sites of evaluation. Overall, the correlation of intensity and lightness was significantly high (*p* < 0.01) in Carillanca and Mafil (Figures 7A,B) with the year of cultivar release, while, for b* and v*, the correlations were significantly high in Santa Rosa and Carillanca (Figures 7C,D).

RGB-derived vegetation indices were negatively (intensity, saturation, lightness, b*, v*, GA, and GGA) and positively (Hue, a*, and u*) correlated with the year of cultivar release and grain yield at the three sites at the booting stage, followed by the anthesis stage (Supplementary Table 2). Only in Carillanca that some of the RGB-derived vegetation indices showed significant correlations with the year of cultivar release (intensity and lightness) and grain yield (intensity, lightness, a*, and GA*) at the grain filling stage.

## Discussion

### Potential Yield and Progress of Winter Wheat in Southern Chile

In this study, the potential yield achieved by winter wheat cultivars in the temperate-humid zone of southern Chile was close to the 20.50 Mg ha^–1^ (Figure 2B) that some authors (Parry et al., 2011; Reynolds et al., 2011; Ray et al., 2012; Hawkesford et al., 2013) have proposed as a target for high-yielding areas. The new cv. Chevignon had the highest yield and regression coefficient (Finlay and Wilkinson slope), indicating greater adaptability (Figure 2A). The prolonged growing period from sowing to the heading of about 180–190 days, a grain filling period of 60–70 days, mild temperatures in December-January, ample water availability (Supplementary Table 1), and favorable soil conditions explain this high potential yield. Indeed, the average photothermal coefficient (Q = solar radiation/mean temperature) during grain filling (15 November-15 January) ranged from 1.40 MJ m^–2^ °C^–1^ at Santa Rosa to 1.78 MJ m^–2^ °C^–1^ at Galvarino.

The historical yield progress observed at Carillanca (a city in the region of Araucania) between 1959 and 2017 resulted from genetic progress and improvements in agronomic practices (Figure 3). The interannual variability detected in grain yield can be explained by changes in environmental conditions, especially precipitation, and the incidence of fungal diseases (e.g., rusts and others since the trials were not sprayed). The highest yield (∼12.90 Mg ha^–1^) was attained in the last 5 years (2013–2017), evidencing the high potential yield of recent cultivars and advanced lines. However, wheat yields attained by farmers are usually lower than the potential yield exhibited by the best-adapted cultivars grown in an experimental field with optimum crop management. Therefore, a yield gap can be estimated by the difference between the potential yield and the average farm yield at a regional level (Lobell et al., 2009; Fischer and Edmeades, 2010). The average yield of the whole Araucania region from 1980 to 1989 was 2.10 Mg ha^–1^, and between 2010 and 2017 was 5.70 Mg ha^–1^ (Oficina de Estudios y Políticas Agrarias [ODEPA], 2020). Compared to the yields obtained at Carillanca for the same period, the yield gap has increased from 3.80 Mg ha^–1^ in the 80s to 5.70 Mg ha^–1^ after 2010. The yield potential of wheat in two high-yielding countries, the United Kingdom and New Zealand, estimated using the Sirius crop model, was between 15.00–19.00 Mg ha^–1^ and 15.60–19.50 Mg ha^–1^ under water-limited and irrigated conditions, respectively (Senapati and Semenov, 2019). The yield gap was estimated at 4.00–6.00 Mg ha^–1^ in both countries, similar to the gap we found between the potential yield and average yield in the Araucania region in southern Chile.

### Genetic Gain of Agronomic Traits

The genetic gain determination is useful to evaluate the progress of crop breeding programs. Studies on yield progress conducted worldwide have reported increases between 0.44 and 1.3% per year (Battenfield et al., 2013); in general, higher yield increases are reported when older and taller cultivars (from before the green revolution) are included in the analysis. In our study, the range of genetic gain in grain yield was 70.20 kg ha^–1^ y^–1^ (0.49%), representing about 55% of the yield progress observed at Carillanca (Figures 3, 4D). Other studies in winter wheat have reported lower or higher genetic gains: 11.00 kg ha^–1^ y^–1^ (0.40%) for semi-dwarf cultivars in low-yielding environments in Grain Plains, United States (Battenfield et al., 2013); 58.00 kg ha^–1^ y^–1^ (1.37%) under irrigation in Turkey for cultivars released between 1963 and 2004 (Gummadov et al., 2015); 57.50 kg ha^–1^ y^–1^ (0.70%) in Henan Province, China, for cultivars released after the 1950s (Gao et al., 2017); 47.40 kg ha^–1^ y^–1^ (0.72%) in the Hebei Province in China for cultivars released between 1964 and 2007 (Yao et al., 2019); and 60.40 kg ha^–1^ y^–1^ (0.72%) under irrigation and 47.50 kg ha^–1^ y^–1^ (0.66%) under rain-fed conditions in England, for cultivars released between 1964 and 2009 (Foulkes et al., 2016). In spring wheat cultivars, the genetic gain in a high-yielding environment (under irrigation) in the Mediterranean zone of Chile has been 43.50 kg ha^–1^ y^–1^ (0.51%) for bread wheat (del Pozo et al., 2014) and 72.80 kg ha^–1^ y^–1^ (0.73%) in durum wheat (del Pozo et al., 2019).

The genetic gains in wheat grain yield since the 1960s (i.e., green revolution) have been achieved by introducing the semi-dwarf genes Rht1 and Rht2, leading to greater partitioning of the shoot biomass to spikes and grains (Shearman et al., 2005; Trethowan et al., 2007; Gummadov et al., 2015). Thus, many agronomic traits have been attributed to these gains, including increased harvest index, thousand kernel weight, number of kernels per spike, Shoot DW, number of spikes per m^2^, and ear length, and decreased plant height (Calderini et al., 1995; Elazab et al., 2021).

Indeed, introducing semi-dwarfing genes in the winter wheat breeding program of INIA in the 1960s reduced plant size from more than 130 cm (cv. Druchamp) to 90–100 in modern cultivars (Table 2). The reduced stem size allows new genotypes to utilize assimilates more efficiently to grow tillers than early genotypes and receive higher N application rates (Rife et al., 2019).

Although the positive correlation was detected between grain yield and Shoot DW in 2019 (Table 2), no genetic gains were reported for Shoot DW (Table 2; Figure 4C). Moreover, the increase in grain yield was associated negatively with the number of spikes per m^2^ (Table 4), and the plant height showed negative gains with the year of cultivar release (Figure 4B). Something similar has been reported for bread wheat varieties released in Argentina between 1918 and 2011 (Lo Valvo et al., 2018), and for winter wheat varieties released in Northern China between 1960 and 2000 (Zhou et al., 2007), but other studies have reported increases in the aboveground biomass with the year of cultivar release (e.g., Xiao et al., 2012; Gao et al., 2017).

The yield progress of winter wheat cultivars released after the 1960s was associated with increases in the number of kernels per spike and the harvest index (Table 2; Figures 4E,F). Indeed, comparing the oldest cv. Druchamp (1965) with the modern cv. Kiron (2017), the increases in the number of kernels per spike and Harvest index were, on average, 43 and 75%, respectively. Increases in the number of kernels per spike after the 1960s have also been reported by other authors (Zheng et al., 2011; Xiao et al., 2012). In our study, genetic gain in the harvest index was observed until 1993, and no further increase has been observed after (Figure 4E). In spring wheat cultivars released by CIMMYT between 1966 and 2009 in the irrigated, high-potential environment of northwest Mexico, there was no increase in harvest index (Aisawi et al., 2015), but other studies performed in high-yielding environments have reported continuing increases in harvest index since the 1960s (Xiao et al., 2012; Lo Valvo et al., 2018; Yao et al., 2019). Modern cultivars are now in the range of 0.45–0.5 (Shearman et al., 2005; Zhou et al., 2007), but further increases are still possible to the theoretical limit of ∼0.6 calculated by Austin et al. (1980). The highest values of the harvest index (0.41) were obtained in cvs. Maxwell (2012) in 2018 and 2019 and Kumpa (2002) in 2018 (Table 2). Reynolds et al. (2011) pointed out that the increase in harvest index might be achieved by optimizing the partitioning of assimilates to different plant organs and increasing spike fertility.

Although significant genotypic effects were found for thousand kernels weight, with cvs. Melifen (1974) and Tukan (1993) presented the lowest and highest thousand kernels weight, the correlation with the year of cultivar release was not significant (Table 4). Nevertheless, the correlation between grain yield and thousand kernels weight for all genotypes across sites was positive and significant in both years (Table 4). Similar studies in winter wheat have reported no changes in thousand kernels weight with the year of release (Xiao et al., 2012), but others have reported a positive relationship (Zhou et al., 2007; Gao et al., 2017; Yao et al., 2019).

We divided the genetic progress data into two different periods (1965–1993) and (1993–2019) to understand why no genetic gains were detected in the harvest index after 1993 (Supplementary Table 3). The results were interesting as the genetic gains from 1965 to 1993 were due to increases in harvest index and thousand kernels weight, and decreases in Shoot DW, days to heading, and plant height, while the genetic gains from 1993 to 2019 were due to increased kernels number per m^2^ and decreases in thousand kernels weight and plant height.

Overall, the results of genetic progress trials confirmed that the main cause for genetic gains detected in grain yield is the increase in the partitioning of biomass toward reproductive organs, without a significant role for increases in shoot biomass production (Austin et al., 1980; Deckerd et al., 1985; Siddique et al., 1989; Slafer et al., 1990; Calderini et al., 1995; Royo et al., 2007; Tshikunde et al., 2019).

### Genetic Gain of Morphophysiological Traits

During grain filling, leaves of the canopy bottom are senescenced, while the flag leaf and the penultimate leaf remain green for a longer time. Thus, the flag leaf is the main photosynthetic organ to support assimilates to the grain, besides the non-foliar tissues (i.e., spikes) and redistribution of assimilates stored in the stem (Simkin et al., 2020; Elazab et al., 2021; Tambussi et al., 2021).

The current study showed significant genotypic differences in An and *gs*, but there was no significant correlation with the year of cultivar release (Table 5). Positive and significant correlations were found between grain yield of genotypes across environments and An (*r* = 0.55; *p* < 0.01) and *gs* (*r* = 0.64, *p* < 0.01) in 2019, but not in 2018 (Table 5). Since yield and biomass accumulation are determined by the integration of various metabolic processes, where measurement of the photosynthesis in the leaf represents a small area at a specific moment (Gutiérrez-RodrıìGuez et al., 2000; Faralli and Lawson, 2020), this might be the reason why we did not always find high correlations between An and grain yield as in the 2018 example. However, there are many examples where improvement in the photosynthetic capacity led to increases in grain yield in wheat cultivars, which could be an option for further improvement of yields in high-yielding environments. For example, significant genetic gains were reported in An at the heading stage (Sun et al., 2014) and, also, after anthesis with 0.70–6.80% per year for facultative wheat released between 1981 and 2008 in Henan Province (Zheng et al., 2011); 0.47% per year for winter wheat cultivars used between 1969 and 2006 in Shandong Province (Xiao et al., 2012); 0.21% per year in ten cultivars released between 1940 and 2009 in Brazil (Beche et al., 2014); and 0.18% per year for winter wheat released between 1964 and 2007 in Hebei Province (Yao et al., 2019).

An and *gs* correlated positively in both years (*r* = 0.35, *p* < 0.05 in 2018; and *r* = 0.40, *p* < 0.01 in 2019; data not shown). However, the reducing trends in the flag leaf area and the specific leaf area in 2019 with the year of cultivar release and the opposite response of chlorophyll content (Table 5 and Figure 5D) supported the idea that the positive association between grain yield and An in 2019 was not only due to higher gs but also due to a higher photosynthetic capacity as well. The lower specific leaf area results in a higher assimilation rate due to increased photosynthetic machinery (i.e., chlorophyll content) per unit leaf area (Sharma-Natu and Ghildiyal, 2005). Furthermore, the positive correlation of chlorophyll content with the year of release (Table 5 and Figure 5D) may reflect more delayed senescence (stay-green) of modern cultivars. Yao et al. (2019) found an increase of 0.17% in chlorophyll content (determined as SPAD index) with the year of cultivar release, and other authors found similarly significant genetic changes in chlorophyll content (Xiao et al., 2012; Balota et al., 2017). Previous studies reported positive and significant correlations between chlorophyll content and grain yield, and Shoot DW and number of kernels per spike, indicating that improvements in chlorophyll content would benefit the genetic gain in grain yield (Yao et al., 2019).

### Genetic Gains of Spectral and Digital RGB-Derived Vegetation Indices

The negative genetic gains of the fraction of intercepted PAR (*fi*) by the crop (Figure 5B) could be due to the more erectophile leaf habit (vertical leaf angle) of modern cultivars compared to older ones (Sadras et al., 2016), which increases the efficiency of intercepting radiation (Siddique et al., 1989). In dense canopies with *fi* > 0.8 (or leaf area index > 3), as in the case of our study (Figures 5A,B), the vertical leaf angles will increase photosynthetic activity (Loomis and Williams, 1969; Stöckle and Kemanian, 2009). The horizontal angle of the leaves increases the extinction coefficient, leading to shading to the bottom part of the canopy, which leads to drying of the bottom leaves, and thus reductions in photosynthetic activity (Thorne, 1971; Borojevic and Kraljevic-Balalic, 1984; Tan et al., 2020). In contrast, the vertical leaf angle facilitates sunlight penetration to the bottom of the canopy, thus enhancing photosynthesis activity by allowing greater light access to a larger proportion of the canopy (Loomis and Williams, 1969; Stöckle and Kemanian, 2009). Such an erectophile leaf habit seemed to be a vital trait for enhancing photosynthetic activity and extending the grain yield performance in wheat.

Like the *fi*, negative genetic gains were detected for the NDVI with the year of cultivar release (Figure 5C). Such a negative association could be due to the nature of the NDVI formulation, which uses the NIR reflectance in its formulation. There are many factors that may cause artefactual decreases in the measured NIR reflectance signal (Gitelson et al., 2002; Elazab et al., 2015, 2016) such as: (1) the canopy architecture, where canopies with erectophile leaves generally scatter more radiation into lower leaf layers than planophile canopies, and, thus, more radiation is trapped within the canopy, increasing the ability of the plant to absorb more light, and, thereby, the NIR reflectance decreases (Gitelson et al., 2002; Elazab et al., 2016); (2) increasing soil moisture content causes a decrease in NIR reflectance of the soil, which may lead to a decrease in the NIR reflectance from the total canopy (Kanemasu, 1974; Huete et al., 1985; Gitelson et al., 2002; Elazab et al., 2016); and (3) transformation of the color of wheat lower leaves from green to brown due to chlorophyll loss (i.e., senescence), which starts from the booting stage, and, thus, the active reflecting leaf layer area decreases, and the consequent NIR reflectance decreases (Leamer et al., 1980; Gitelson et al., 2002).

The Shoot DW in the present study did not show any genetic gains with the year of cultivar release (Figure 4C). Both the Shoot DW determinants, the accumulated intercepted PAR (IPAR), and the RUE showed no genetic gains with the year of cultivar release (*r*^2^ = 0.03, *p* > 0.05 for IPAR from emergence to maturity, figure not shown; *r*^2^ = 0.06, *p* > 0.05 for RUE, Figure 6B). Moreover, the IPAR and RUE did not show significant correlations with Shoot DW (*r*^2^ = 0.006, *p* > 0.05 for IPAR; *r*^2^ = 0.28, *p* > 0.05 for RUE; figures not shown). These results are in agreement with the studies of Deckerd et al. (1985) and Slafer et al. (1990), who suggested that the Shoot DW did not show any trend with the year of cultivar release because either the IPAR or the RUE changed in opposite directions and/or both traits have no association with Shoot DW. The correlations between photosynthetic capacity and grain yield might not necessarily be straightforward for modern wheat genotypes, which are reported to be source limited (Reynolds et al., 2000a). This source limitation is because the amount of CO_2_ fixed for plant growth is reduced by physiological processes, such as the respiration of assimilates during the dark period, as well as the loss of carbon from root exudates, senescence, and other processes (Amthor, 1989; Reynolds et al., 2000b).

The negative association detected between IPAR calculated from sowing to heading and the year of cultivar release (Figure 6A) is mainly due to the longer growing period required to reach heading in older cultivars (Figure 4A), as positive associations have been detected between DH and IPAR from sowing to heading (*r* = 0.86; *p* < 0.0001, figure not shown). The positive genetic gains detected for IPAR calculated from heading to maturity (Figure 6A) with the year of cultivar release are mainly due to the extended period from heading to maturity, which increased the total amount of incident radiation, and not due to enhanced *fi*, which showed negative correlations with the year of cultivar release (see Figure 5B). The extended flag leaf duration may partially play a role in the positive genetic gains detected for IPAR with the year of cultivar release. After heading, most of the dry weight of the wheat grains is mainly derived from CO_2_ taken up by parts of the canopy located above the flag leaf node (Thorne, 1965). Moreover, previous studies have reported that enhanced flag leaf duration (Fischer and Kohn, 1966; Simón, 1999; Foulkes et al., 2016) and ear photosynthesis (Maydup et al., 2010, 2012, 2014; Merah and Monneveux, 2015; Merah et al., 2018; Elazab et al., 2021) are correlated with grain yield. The flag leaf chlorophyll content from heading until grain filling (Table 5 and Figure 5D) showed a weak but significant association with the year of cultivar release. Furthermore, Elazab et al. (2021) reported a role for ear photosynthesis during grain filling for the same set of genotypes.

Like both the *fi* and NDVI, most RGB-derived vegetation indices (except Hue, a*, and u*) showed negative correlations with the year of release and grain yield, especially at the booting stage, followed by the heading stage (Supplementary Table 2 and Figure 7). Casadesús et al. (2007); Casadesús and Villegas (2014), Vergara-Díaz et al. (2016); Kefauver et al. (2017), and Fernandez-Gallego et al. (2019) using the same digital RGB-derived vegetation indices have shown opposite correlations (i.e., positive correlations) with grain yield in wheat and maize at different growth stages (tillering, booting, heading, anthesis, and grain filling). A positive correlation between these RGB-derived vegetation indices and grain yield is expected when a positive correlation is detected between Shoot DW and grain yield (Casadesús et al., 2007), but this was not fully accomplished in our study as discussed before.

The three locations showed that the booting stage is the best phenological stage for detecting variability in grain yield (Supplementary Table 2). The weak correlations after the booting stage could be due to several disturbance factors, such as the appearance of productive organs of lower chlorophyll content (i.e., spikes) (Tambussi et al., 2007), the previously mentioned erectophile architecture of the canopy, and the senescence of bottom canopy leaves (starting from booting), which may decrease the ability of the RGB-derived vegetation indices to capture the characteristics of the green biomass after the booting stage.

In general, explaining the grain yield and shoot biomass relationship using the spectral and/or digital RGB-derived vegetation indices is not always straightforward (Casadesús et al., 2007). The vegetation index response could vary in stressed environments according to stress timing and severity (Bort et al., 2005), such as in Mediterranean regions where terminal water and/or heat stress during the grain-filling period are common (López-Castañeda and Richards, 1994; del Pozo et al., 2016; Royo et al., 2018). Thus, low values and even negative associations of the vegetation index, especially during grain filling, could indicate a shorter crop cycle or an avoidance mechanism from the stress conditions (Bort et al., 2005; Casadesús et al., 2007). Overall, the studied spectral and RGB-derived vegetation indices confirmed a minor role for Shoot DW production in the genetic gains detected in grain yield, as confirmed previously by the yield components (Tables 2, 4; Figure 4).

## Conclusion

The yield potential of winter wheat in southern Chile is very high, reaching 20.46 Mg ha^–1^ at the most favorable sites. The prolonged growing period from sowing to the heading of about 180–190 days, a grain filling period of 60–70 days, mild temperatures in December-January, ample water availability, and favorable soil conditions explain this high potential yield. The yield progress trail showed that grain yield has increased over the last 60 years from 2.7 Mg ha^–1^ in 1959 to 12.9 Mg ha^–1^ in 2017, with an annual increase of 128.8 kg ha^–1^ per year. The genetic gain in grain yield from 1965 and 2019 has been 70.20 kg ha^–1^ (0.49%) per year, representing around 55% of the yield progress. Our results confirmed that the main cause of the genetic gains detected in grain yield is the increased biomass partitioning toward reproductive organs, without a significant role for increases in shoot biomass production. The changes in the plant morphophysiological during the past 60 years appeared to play a significant role in the detected grain yield gains by: (1) the reducing trends in the flag leaf area and the specific leaf area with the year of cultivar release, while the opposite trend was detected for chlorophyll content, which confirmed a higher photosynthetic capacity as the lower specific leaf area results in a higher assimilation rate due to the increase in in the amount of photosynthetic machinery (i.e., chlorophyll content) per unit leaf area; (2) The negative relationship between the year of cultivar release and the NDVI, *fi*, IPAR (from emergence and heading), and the RGB-derived vegetation indices (intensity, lightness, b*, and v*), which could be attributed to the erectophile leaf habit, which seemed to be a vital trait for enhancing photosynthetic activity and extending the grain yield performance in wheat, and, also, the senescence of bottom canopy leaves (starting from booting), which may decrease the ability of the spectral and RGB-derived vegetation indices to capture the characteristics of the green biomass after the booting stage; and (3) The positive trends detected for IPAR (from heading to maturity) with the year of cultivar release, which could be due to a stay-green mechanism, which is supported by the trend of positive correlations of chlorophyll content with the year of release.

## Data Availability Statement

The original contributions presented in the study are included in the article/Supplementary Material, further inquiries can be directed to the corresponding author.

## Author Contributions

AP, CJ, and IM designed the field experiments. CJ provided all winter wheat genotypes and the historical data of grain yield from Carillanca. CJ, IM, and DC evaluated agronomic traits. AP, AM-E and MG evaluated physiological traits. AP and AE analyzed RGB-derived indices. AP, AM-E, and AE performed the statistical analysis. AP was in charge of the writing. All authors contributed to the manuscript, read, and approved the final manuscript.

## Conflict of Interest

The authors declare that the research was conducted in the absence of any commercial or financial relationships that could be construed as a potential conflict of interest.

## Publisher’s Note

All claims expressed in this article are solely those of the authors and do not necessarily represent those of their affiliated organizations, or those of the publisher, the editors and the reviewers. Any product that may be evaluated in this article, or claim that may be made by its manufacturer, is not guaranteed or endorsed by the publisher.

## Footnotes

1
https://www.pix4d.com/

2
https://gitlab.com/sckefauver/cerealscanner

3
http://fiji.sc/Fiji

4
http://dynamax.com/images/uploads/papers/SS1_Manual.pdf
