# Supplementary material for: Genetic Yield Gains and Changes in Morphophysiological-Related Traits of Winter Wheat in Southern Chilean High-Yielding Environments
Source: Front Plant Sci. 2022 Jan 3;12:732988. doi: 10.3389/fpls.2021.732988 (PMC8761861; doi:10.3389/fpls.2021.732988)
Supplement: Supplementary file 1 [file Table_1.DOCX]

**Supplementary Table S1**| Mean monthly and annual temperatures and solar radiation, and monthly and annual total rainfall (mm) at different sites in central-southern Chile.

**A)** Santa Rosa, Ñuble Region (36°31’ S; 71°54’ W)

|  | Year | Jan | Feb | Mar | Apr | May | Jun | Jul | Aug | Sep | Oct | Nov | Dec | Annual |
| --- | --- | --- | --- | --- | --- | --- | --- | --- | --- | --- | --- | --- | --- | --- |
| Temp | 2018 | 19.5 | 19.3 | 15.4 | 11.5 | 9.1 | 6.0 | 6.6 | 7.6 | 10.9 | 12.0 | 15.4 | 18.0 | 12.6 |
| (°C) | 2019 | 18.9 | 19.7 | 15.7 | 12.0 | 9.7 | 7.9 | 7.9 | 8.3 | 9.6 | 12.1 | 16.1 | 18.5 | 13.0 |
| Radiation | 2018 | 25.8 | 24.5 | 19 | 11.1 | 6.8 | 5.6 | 7.2 | 9.7 | 13.3 | 17.3 | 22.5 | 25.9 | 15.7 |
| (MJ m^-2^) | 2019 | 25.4 | 21.8 | 17.4 | 10.8 | 6.2 | 5.0 | 6.7 | 9.5 | 13.3 | 17.6 | 22.3 | 25.1 | 15.1 |
| Rainfall | 2018 | 4.9 | 7.1 | 30.9 | 73.8 | 85.7 | 118 | 101.3 | 92.8 | 102.2 | 97.2 | 64.8 | 15.3 | 794 |
| (mm) | 2019 | 9.1 | 7.8 | 8.2 | 7.4 | 140.6 | 221.6 | 93.9 | 52.1 | 57.3 | 15.1 | 12.6 | 5.8 | 632 |

**B)** Yungay, Ñuble Region (37°14´ S, 72°01´ W)

|  | Year | Jan | Feb | Mar | Apr | May | Jun | Jul | Aug | Sep | Oct | Nov | Dec | Annual |
| --- | --- | --- | --- | --- | --- | --- | --- | --- | --- | --- | --- | --- | --- | --- |
| Temp | 2018 | 19.5 | 19.9 | 15.8 | 12.6 | 9.5 | 6.9 | 7.4 | 8 | 10.8 | 11.7 | 14.9 | 17.7 | 12.9 |
| (°C) | 2019 | 18.8 | 20.2 | 16.3 | 13.2 | 10.6 | 8.3 | 8.2 | 8.6 | 9.8 | 11.9 | 15.8 | 18.2 | 13.3 |
| Radiation | 2018 | 24.3 | 23.4 | 18.1 | 11.3 | 5.6 | 4.9 | 6.0 | 8.1 | 12.0 | 16.1 | 22.2 | 25.8 | 14.8 |
| (MJ m^-2^) | 2019 | 25 | 20.5 | 17.2 | 10.4 | 6.0 | 3.9 | 5.2 | 7.9 | 12.3 | 16 | 21.6 | 25.2 | 14.3 |
| Rainfall | 2018 | 9.9 | 14.1 | 26.5 | 121.3 | 113.3 | 174.5 | 74 | 101.7 | 165.7 | 110.6 | 130.9 | 24.5 | 1067 |
| (mm) | 2019 | 31.5 | 2.2 | 16.5 | 10.1 | 166.2 | 339.5 | 137.1 | 78.9 | 56.6 | 38.1 | 16.8 | 12.7 | 906.2 |

**C)** Humán, Biobio Region (37°.43° S; 72°24’ W)

|  | Year | Jan | | Feb | | Mar | Apr | | May | | Jun | | Jul | | Aug | Sep | | Oct | | Nov | | Dec | Annual | |  |  |  |  |
| --- | --- | --- | --- | --- | --- | --- | --- | --- | --- | --- | --- | --- | --- | --- | --- | --- | --- | --- | --- | --- | --- | --- | --- | --- | --- | --- | --- | --- |
| Temp | 2018 | 19.0 | | 19.9 | | 15.5 | 11.6 | | 9.9 | | 6.7 | | 7.1 | | 8.3 | 10.7 | | 11.6 | | 14.7 | | 17.1 | 12.7 | |  |  |  |  |
| (°C) | 2019 | 18.5 | | 20.2 | | 16.5 | 13.0 | | 9.9 | | 7.3 | | 8.0 | | 8.6 | 10.0 | | 11.7 | | 15.3 | | 18.4 | 13.1 | |  |  |  |  |
| Radiation | 2018 | 25.3 | | 23.7 | | 18.1 | 11.0 | | 6.7 | | 5.6 | | 7.0 | | 8.9 | 13.1 | | 17.6 | | 23.1 | | 27.2 | 15.6 | |  |  |  |  |
| (MJ m^-2^) | 2019 | 26.9 | | 22.1 | | 18.9 | 10.5 | | 6.9 | | 5.6 | | 5.7 | | 8.8 | 14 | | 17.3 | | 22.9 | | 25.6 | 15.4 | |  |  |  |  |
| Rainfall | 2018 | 17.9 | | 28.3 | | 77.2 | 137.3 | | 179.1 | | 141.2 | | 71.8 | | 107.0 | 201.1 | | 71.9 | | 80.3 | | 26.7 | 1139 | |  |  |  |  |
| (mm) | 2019 | 24.4 | | 1.4 | | 16.7 | 10.0 | | 38.4 | | 98.1 | | 113.5 | | 70.4 | 32.4 | | 58.8 | | 11.2 | | 15.7 | 491 | |  |  |  |  |
| **D)** Curacautin, Araucanía (38.40°31’ S; 71°90’ W) | | | | | | | | | | | | | | | | | | | | | | | | | | | | |
|  | Year | | Jan | | Feb | | | Mar | | Apr | | May | | Jun | | | Jul | | Aug | | Sep | | | Oct | | Nov | Dec | Annual |
| Temp | 2018 | | 15 | | 16 | | | 11.6 | | 9.1 | | 8.1 | | 4.7 | | | 5.2 | | 6.1 | | 7.4 | | | 9 | | 11.4 | 14 | 9.8 |
| (°C) | 2019 | | 13.6 | | 16.9 | | | 12.9 | | 10.2 | | 8.4 | | 6.4 | | | 5.8 | | 6.4 | | 7.1 | | | 8.9 | | 12 | 13 | 10.1 |
| Radiation | 2018 | | 23.2 | | 21.4 | | | 14.8 | | 9.6 | | 6.4 | | 4.7 | | | 6 | | 7.8 | | 11.3 | | | 15 | | 20.3 | 23.9 | 13.7 |
| (MJ m^-2^) | 2019 | | 23.2 | | 20.7 | | | 16.8 | | 10.2 | | 6.2 | | 4.3 | | | 5.3 | | 8.5 | | 12.1 | | | 16.8 | | 20.1 | 21.1 | 13.8 |
| Rainfall | 2018 | | 41.7 | | 42.1 | | | 163 | | 195.1 | | 213.3 | | 288.4 | | | 105 | | 136.4 | | 267.8 | | | 135.5 | | 219.7 | 48.3 | 1856 |
| (mm) | 2019 | | 74.2 | | 20.6 | | | 27.7 | | 62.7 | | 196.8 | | 375.2 | | | 273.2 | | 119.8 | | 98 | | | 129 | | 64.2 | 47 | 1488 |

**E)** Galvarino, Araucania Region (38.45°31’ S; 72°74’ W)

|  | Year | Jan | Feb | Mar | Apr | May | Jun | Jul | Aug | Sep | Oct | Nov | Dec | Annual |
| --- | --- | --- | --- | --- | --- | --- | --- | --- | --- | --- | --- | --- | --- | --- |
| Temp | 2018 | 16.6 | 17.6 | 13.7 | 10.7 | 9.9 | 6.5 | 6.7 | 7.4 | 9.1 | 10.4 | 12.2 | 14.9 | 11.3 |
| (°C) | 2019 | 15.7 | 18.0 | 14.8 | 12.1 | 10.0 | 8.2 | 8.0 | 7.9 | 8.8 | 10.7 | 13.2 | 15.0 | 11.9 |
| Radiation | 2018 | 26.7 | 23 | 17.0 | 10.1 | 6.5 | 4.4 | 6.0 | 7.4 | 11.8 | 17 | 22.3 | 25.7 | 14.8 |
| (MJ m^-2^) | 2019 | 27.1 | 22.5 | 17.0 | 10.0 | 6.0 | 4.8 | 5.7 | 8.2 | 12.7 | 18.5 | 22.8 | 26.7 | 15.2 |
| Rainfall | 2018 | 11.1 | 16.6 | 118.5 | 112.4 | 131.6 | 108.8 | 39.4 | 96.6 | 55.4 | 46.1 | 61.8 | 35.7 | 834 |
| (mm) | 2019 | 7.0 | 3.2 | 14.9 | 20.0 | 93.0 | 206.0 | 173.8 | 57.2 | 41.6 | 36.6 | 20.6 | 5.9 | 679 |

**F)** Carillanca, Araucania Region (38°69´ S, 72°41´ W)

|  | Year | Jan | Feb | Mar | Apr | May | Jun | Jul | Aug | Sep | Oct | Nov | Dec | Annual |
| --- | --- | --- | --- | --- | --- | --- | --- | --- | --- | --- | --- | --- | --- | --- |
| Temp | 2018 | 15.9 | 16.3 | 12.9 | 9.8 | 8.8 | 5.6 | 6 | 6.7 | 8.5 | 10.1 | 12.1 | 14.4 | 10.6 |
| (°C) | 2019 | 14.5 | 16.7 | 13.3 | 10.9 | 8.9 | 7.5 | 7.1 | 7.2 | 8.4 | 10.2 | 12.8 | 14.4 | 11.0 |
| Radiation | 2018 | 23.7 | 20.2 | 14.9 | 9.5 | 5.7 | 3.8 | 5.3 | 6.7 | 10.1 | 15.2 | 20.4 | 23.6 | 13.3 |
| (MJ m^-2^) | 2019 | 24.2 | 20.9 | 15.5 | 8.0 | 5.2 | 4.5 | 5.2 | 7.6 | 11.9 | 16.4 | 20.9 | 24.6 | 13.7 |
| Rainfall | 2018 | 20.7 | 19.6 | 130 | 170 | 161.2 | 229.3 | 88.9 | 135.6 | 152.7 | 161.1 | 89.4 | 42.4 | 1400 |
| (mm) | 2019 | 16.1 | 6.4 | 26.1 | 21.5 | 118.3 | 285.6 | 221.2 | 94.3 | 53.1 | 41.8 | 30.4 | 9.9 | 925 |

**G)** Mafil, Los Ríos Region (39°70 S; 73°01’ W)

|  | Year | Jan | Feb | Mar | Apr | May | Jun | Jul | Aug | Sep | Oct | Nov | Dec | Annual |
| --- | --- | --- | --- | --- | --- | --- | --- | --- | --- | --- | --- | --- | --- | --- |
| Temp | 2018 | 16.7 | 17.3 | 13.5 | 10.4 | 9.6 | 6.5 | 6.1 | 7.3 | 8.9 | 10.2 | 12.6 | 15.4 | 11.2 |
| (°C) | 2019 | 15.2 | 17.2 | 14.1 | 11.2 | 9.8 | 8.3 | 7.5 | 7.2 | 8.6 | 10.2 | 13.1 | 15.1 | 11.4 |
| Radiation | 2018 | 23.1 | 20.2 | 13.8 | 8.9 | 5.2 | 3.5 | 4.3 | 6.1 | 9.9 | 13.5 | 17.3 | 21.4 | 12.3 |
| (MJ m^-2^) | 2019 | 21.4 | 19.5 | 14.4 | 7.6 | 4.0 | 3.7 | 3.9 | 5.8 | 10.8 | 15.1 | 19.0 | 25.9 | 12.6 |
| Rainfall | 2018 | 41.9 | 13.1 | 202 | 210.3 | 142.5 | 223.7 | 126.9 | 325.4 | 182.5 | 117.5 | 94.4 | 48.4 | 1729 |
| (mm) | 2019 | 18.0 | 7.7 | 21.4 | 46.3 | 235.2 | 261.1 | 360.7 | 122.6 | 55.1 | 80.6 | 64.6 | 28.9 | 1302 |

**H)** Purranque, Los Lagos Region (40°86; 73°15)

|  | Year | Jan | Feb | Mar | Apr | May | Jun | Jul | Aug | Sep | Oct | Nov | Dec | Annual |
| --- | --- | --- | --- | --- | --- | --- | --- | --- | --- | --- | --- | --- | --- | --- |
| Temp | 2018 | 14.9 | 15.8 | 12.5 | 9.7 | 8.8 | 5.7 | 5.4 | 6.9 | 8.5 | 9.8 | 11.3 | 13.9 | 10.3 |
| (°C) | 2019 | 13.1 | 15.5 | 13.0 | 10.2 | 8.7 | 7.2 | 6.7 | 6.8 | 8.0 | 9.7 | 11.9 | 13.4 | 10.4 |
| Radiation | 2018 | 24.6 | 20.9 | 14.6 | 9.7 | 6.0 | 4.0 | 5.4 | 8.2 | 11.7 | 15.6 | 19.1 | 22.8 | 13.6 |
| (MJ m^-2^) | 2019 | 23.8 | 21.8 | 15.6 | 8.7 | 5.0 | 4.9 | 5.0 | 7.1 | 12.3 | 16.6 | 19.6 | 23.3 | 13.6 |
| Rainfall | 2018 | 27.4 | 14.4 | 142.9 | 109.1 | 120.3 | 105.1 | 62.9 | 193.9 | 132.4 | 119.6 | 73.9 | 39.9 | 1142 |
| (mm) | 2019 | 26.1 | 16.0 | 10.9 | 40.8 | 102.9 | 181.4 | 220 | 104.4 | 40.2 | 63.6 | 93.1 | 44.8 | 944 |
