# Supplementary material for: Genetic Yield Gains and Changes in Morphophysiological-Related Traits of Winter Wheat in Southern Chilean High-Yielding Environments
Source: Front Plant Sci. 2022 Jan 3;12:732988. doi: 10.3389/fpls.2021.732988 (PMC8761861; doi:10.3389/fpls.2021.732988)
Supplement: Supplementary file 2 [file Table_2.DOCX]

**Supplementary Table S2**| Pearson correlation coefficients between RGB-derived vegetation indices evaluated in November-December and year of cultivar release **(A)** and grain yield **(B)** at Santa Rosa, Carillanca, and Mafil in 2019. Values in bold are significant (*P < 0.05 and **P < 0.01). The developmental stages for the three evaluations were booting (Z4.7), heading (Z5.9), and grain filling (Z7.7, late milk). The Pearson correlations of year of release and grain yield were performed using best linear unbiased estimator (BLUE) value of across cultivars in each site.

| **A)** Year of cultivar release | |  |  |  |  |  |  |  |  |  |  |
| --- | --- | --- | --- | --- | --- | --- | --- | --- | --- | --- | --- |
|  | Santa Rosa | | |  | Carillanca | | |  | Máfil | | |
| RGB indices | Booting | Heading | Grain filling |  | Booting | Heading | Grain filling |  | Booting | Heading | Grain filling |
| Intensity | **-0.76^**^** | -0.44 | 0.41 |  | **-0.75^**^** | **-0.62^*^** | **0.56^*^** |  | **-0.85^**^** | **-0.78^**^** | 0.28 |
| Hue | **0.62^*^** | 0.07 | -0.24 |  | **0.76^**^** | -0.35 | -0.19 |  | **0.65^*^** | 0.37 | -0.08 |
| Saturation | **-0.58^*^** | -0.31 | -0.36 |  | 0.02 | 0.28 | -0.43 |  | -0.52 | **0.64^*^** | -0.12 |
| Lightness | **-0.79^**^** | **-0.55^*^** | 0.36 |  | **-0.78^**^** | **-0.63^*^** | **0.54^*^** |  | **-0.84^**^** | **-0.79^**^** | 0.24 |
| a* | **0.90^**^** | **0.78^**^** | 0.46 |  | 0.33 | 0.41 | 0.44 |  | **0.69^**^** | -0.09 | 0.39 |
| b* | **-0.91^**^** | **-0.73^**^** | -0.28 |  | **-0.80^**^** | -0.14 | -0.36 |  | -0.47 | -0.38 | -0.07 |
| u* | **0.73^**^** | **0.67^**^** | 0.37 |  | 0.10 | 0.49 | 0.28 |  | **-0.71^**^** | -0.25 | 0.27 |
| v* | **-0.89^**^** | **-0.77^**^** | -0.34 |  | **-0.85^**^** | -0.31 | -0.35 |  | **-0.63^*^** | -0.47 | -0.07 |
| GA | **-0.83^**^** | -0.45 | -0.49 |  | -0.41 | -0.33 | -0.43 |  | 0.00 | 0.06 | -0.01 |
| GGA | -0.41 | **-0.54^*^** | -0.47 |  | -0.46 | -0.42 | -0.28 |  | 0.25 | 0.41 | -0.09 |
|  |  |  |  |  |  |  |  |  |  |  |  |
| **B)** Grain yield |  |  |  |  |  |  |  |  |  |  |  |
|  | Santa Rosa | | |  | Carillanca | | |  | Máfil | | |
| RGB indices | Booting | Heading | Grain filling |  | Booting | Heading | Grain filling |  | Booting | Heading | Grain filling |
| Intensity | **-0.54^*^** | **-0.60^*^** | -0.14 |  | **-0.52*** | -0.29 | **0.72^**^** |  | **-0.57^*^** | -0.43 | -0.14 |
| Hue | 0.52 | -0.16 | -0.04 |  | **0.64^*^** | -0.35 | -0.36 |  | **0.57^*^** | -0.05 | 0.02 |
| Saturation | -0.47 | 0.13 | -0.13 |  | -0.26 | -0.01 | -0.53 |  | 0.13 | 0.24 | -0.05 |
| Lightness | **-0.56*** | **-0.62^*^** | -0.19 |  | **-0.56^*^** | -0.36 | **0.70^**^** |  | **-0.58^*^** | -0.41 | -0.13 |
| a* | **0.57^*^** | 0.50 | 0.16 |  | 0.45 | **0.61^*^** | **0.60^*^** |  | -0.50 | -0.16 | -0.16 |
| b* | **-0.66^*^** | -0.37 | -0.39 |  | **-0.77^**^** | -0.30 | -0.45 |  | -0.47 | 0.04 | -0.01 |
| u* | 0.38 | 0.50 | 0.08 |  | 0.23 | **0.64^*^** | 0.40 |  | **0.60^*^** | -0.06 | -0.14 |
| v* | **-0.64^*^** | -0.48 | -0.46 |  | **-0.76^**^** | -0.40 | -0.44 |  | **-0.55^*^** | -0.02 | -0.01 |
| GA | -0.48 | -0.10 | -0.22 |  | -0.35 | -0.29 | **-0.62^*^** |  | 0.04 | 0.34 | 0.35 |
| GGA | 0.05 | -0.19 | -0.14 |  | -0.35 | -0.43 | -0.44 |  | 0.47 | 0.49 | 0.29 |
