# Supplementary material for: Genetic Yield Gains and Changes in Morphophysiological-Related Traits of Winter Wheat in Southern Chilean High-Yielding Environments
Source: Front Plant Sci. 2022 Jan 3;12:732988. doi: 10.3389/fpls.2021.732988 (PMC8761861; doi:10.3389/fpls.2021.732988)
Supplement: Supplementary file 3 [file Table_3.docx]

**Supplementary Table S3**| Pearson correlations of year of cultivar release with the studied agronomic traits.

|  | 1965-1993 | 1993-2019 |
| --- | --- | --- |
| Days to heading | -0.48^**^ | 0.06 |
| Plant height | -0.53^**^ | -0.46^**^ |
| Grain yield | 0.48^**^ | 0.39^**^ |
| Shoot DW | -0.32^*^ | 0.28 |
| Harvest index | 0.67^**^ | 0.09 |
| Number of spikes per m^2^ | -0.13 | 0.23 |
| Number of kernels per spike | 0.10 | 0.26 |
| Thousand kernels weight | 0.55^**^ | -0.43^**^ |
| Number of Kernels per m^2^ | -0.01 | 0.36^*^ |

The Pearson correlations were performed using BLUE values for each site of the two years of evaluation.

*: P < 0.05; and **: P < 0.01.
